# Recent Advances in Laser‐Induced Graphene‐Based Gas Sensors: From Sensing Mechanisms to Biomedical Applications

**DOI:** 10.1002/advs.202521138

**Published:** 2026-01-30

**Authors:** Md Abu Sayeed Biswas, Ankan Dutta, Shuvendu Das, Abu Musa Abdullah, Wanqing Zhang, Fatema Tuz Zohra, Huanyu Cheng

**Affiliations:** ^1^ Department of Engineering Science and Mechanics The Pennsylvania State University University Park USA; ^2^ Center for Neural Engineering The Pennsylvania State University University Park USA

**Keywords:** input signal decoupling, laser‐induced graphene, porous structures and nanocomposites, standalone sensing platforms, wearable gas sensors

## Abstract

Direct laser irradiation of carbonaceous precursors such as polyimide and wood is a scalable and efficient method to produce a 3D porous graphene structure, noted as laser‐induced graphene (LIG). The electrical and chemical properties of the LIG can be optimized for the target application by mixing or doping with transition metal dichalcogenides or others to form LIG nanocomposites. Fine‐tuning these properties has led to extensive use in sensing various gaseous biomarkers from the human body or the environment. This review first discusses the gas‐sensing mechanism of LIG and its nanocomposites, followed by a summary of different fabrication methods and performance characteristics. It also examines recent developments in soft functional packaging, such as semi‐permeable membranes or hermetic encapsulations, to provide the sensors with moisture‐resistance or signal decoupling capabilities. Despite the efforts to further improve the gas sensing characteristics, the recently developed wireless and standalone gas sensing platforms highlight a different opportunity for the commercialization of wearable gas sensors. Strategies executed by other gas sensors have been proposed to mitigate the problems and challenges faced by the LIG‐based nanocomposite gas sensors. This review serves as a comprehensive reference on LIG‐based nanocomposite gas sensors, from fundamentals of sensing mechanisms to real‐life applications.

## Graphical abstract

Laser‐induced graphene (LIG) provides a scalable, laser‐direct‐written route to porous graphene architecture with tunable chemistry and defect density. Through heterojunction engineering, catalytic functionalization, and intrinsic self‐heating, LIG achieves highly sensitive and selective detection of NO_X_, NH_3_, H_2_, and humidity, supporting next‐generation wearable and standalone gas‐sensing systems.

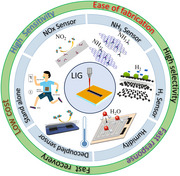

## 1 Introduction

By combining flexibility and adaptability to monitor various physiological and environmental parameters, wearable sensors hold a high potential to serve as an alternative pathway to clinical diagnostics [1, 2, 3, 4, 5, 6, 7]. Wearable sensors comfortably adhere to irregular human body surfaces and can provide real‐time data on vital signs, movements, environmental conditions, and more [8, 9, 10, 11, 12]. They have shown immense promise in applications ranging from healthcare (by tracking heart rate [13, 14, 15], body temperature [16], and sleep patterns [17, 18] to environmental monitoring (by measuring pollution levels and detecting toxic gases [19, 20, 21, 22]. Gas sensors play a pivotal role in human health monitoring by providing early detection and continuous monitoring of harmful gases such as carbon monoxide, nitrogen dioxide, and various volatile organic compounds. These sensors are instrumental in assessing indoor air quality, which is crucial for preventing health issues from poor indoor environments. For individuals with respiratory conditions like asthma, gas sensors could help manage their health by monitoring pollutants that can exacerbate their symptoms. Additionally, gas sensors ensure occupational safety in industrial settings by detecting toxic or flammable gases. Integrating gas sensors into wearable health devices enables real‐time health insights and proactive health management. However, these flexible gas sensors' extensive and intricate preparation procedures have impeded their mass production. Recently, laser‐induced graphene (LIG) featuring a highly porous structure with abundant active sites has emerged as a promising material for gas sensing [23, 24, 25, 26]. While similar to graphene, LIG exhibits distinct material characteristics [21, 22, 27, 28, 29, 30, 31]. However, graphene is a semimetal with a lack of bandgap and can be doped as either p‐type or n‐type by introducing electron acceptors or donors [32, 33, 34]. In contrast, LIG is typically considered a p‐type semiconducting material [35, 36]. This classification is primarily due to LIG's unique interaction with oxygen species when exposed to air. Specifically, LIG has an affinity to adsorb negatively charged oxygen species on its surface, forming a hole accumulation layer (HAL) near the surface. In this context, holes serve as the majority carriers in LIG, while electrons become the minority carriers. This fundamental difference in electronic behavior distinguishes LIG from pristine graphene and plays a pivotal role in its gas‐sensing performance. The synthesis of LIG involves laser irradiation of carbonaceous precursor polymers such as polyimide (PI) [37, 38, 39], polyether sulfone [40, 41], phenolic [42, 43], and even wood [44, 45]. into a porous 3D graphene structure in a single step. Localized heating induced by the laser energy causes the carbonization of non‐carbon elements and functional groups. It facilitates the re‐arrangement of carbon atoms, prevalent in the precursor, from sp^3^ hybridization to sp^2^ hybridization to form few‐layered graphene structures. Laser ablating the carbonaceous polymers induces rapid generation and expansion of vaporized non‐carbon materials to form bubbles that burst, creating voids resulting in a porous 3D graphene structure. LIG synthesis is scalable, cost‐effective, and rapid, and allows for direct patterning without complex masking or processing methods, making it suitable for wearable flexible sensors for human health monitoring and disease diagnosis [46, 47], environment monitoring [48, 49], fuel cells [50, 51], and energy storage devices such as supercapacitors [52, 53].

LIG gas sensors exhibit remarkable versatility, making them promising candidates for various gas detection applications. The ability of LIG gas sensors to detect a group of hazardous air pollutants, such as NOx, with high sensitivity and selectivity has opened up new possibilities for real‐time monitoring of air quality, emissions control, and environmental protection [54]. Beyond NOx detection, LIG gas sensors have demonstrated their effectiveness in detecting various other gases, including ammonia, hydrogen, and humidity. Their tunable surface properties and high surface area‐to‐volume ratio enable the selective adsorption and detection of these gases at extremely low concentrations. Such capabilities are particularly valuable in applications ranging from industrial safety to agricultural monitoring. Additionally, LIG gas sensors possess an inherent self‐heating capability [55, 56, 57], a unique characteristic that eliminates the need for external heating elements to simplify sensor design. This localized self‐heating feature reduces power consumption and enhances sensor response times, making LIG gas sensors highly competitive in terms of energy efficiency and responsiveness. Decoupling multi‐parameter sensors has shown promise as they allow for the simultaneous measurement of multiple input signals at high accuracy, providing a more comprehensive understanding of complex systems. This decoupling of variables enables better control, optimization, and problem‐solving in various applications, from industrial processes to environmental monitoring and healthcare, ultimately leading to improved efficiency and effectiveness. In a recent study, two LIG sensors with encapsulation applied for one and self‐heating applied for the other can decouple gas and temperature to monitor essential agricultural parameters such as fertilizer emission and soil temperature [54]. Along with decoupled sensing, the standalone system has been gaining attention due to its low‐cost and low‐weight characteristics, as it eliminates the need for battery replacement or the usage of a battery completely. In the LIG‐based standalone gas sensor system, the triboelectric nanogenerator could harvest the intermittent kinetic energy into electric energy and store it in the micro supercapacitor array for driving gas sensors for public health monitoring [58].

**FIGURE 1:**
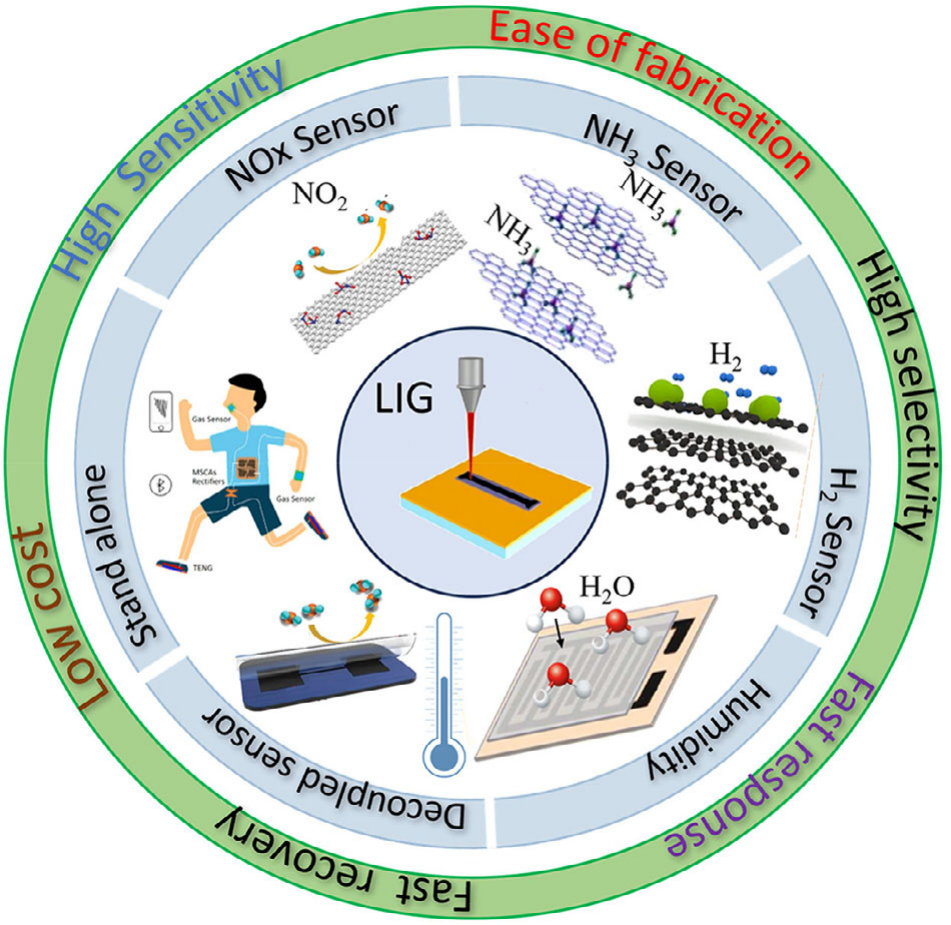
Laser‐induced graphene‐based wearable gas sensors application in human health monitoring.

In this comprehensive review, we will explore the recent developments in LIG gas sensor technology, elucidating the principles of operation, fabrication methods, and performance characteristics across different gas detection applications. The review delves into the gas‐sensing mechanisms of LIG and its nanocomposites, discussing fabrication methods and characteristics such as sensitivity, selectivity, response time, and limit of detection. While efforts continue to enhance gas‐sensing properties, the review highlights the potential of wireless and standalone gas sensing platforms with feasible pathways for future commercialization. It concludes by proposing strategic future prospects of LIG gas sensors, emphasizing their potential to reshape the landscape of gas sensing technology and pave the way for innovative solutions to pressing environmental and human health‐related challenges. Overview of the design and application landscape of LIG‐based wearable gas sensor in healthcare (Figure 1).

## 2 General Gas Sensing Methods

Gas sensing methods rely on various mechanisms depending on sensing materials, transduction principles, and the interaction between active layers and target gas molecules. Optical gas sensors, particularly non‐dispersive infrared (NDIR) sensors, detect gases based on a characteristic absorption peak in the UV–vis or infrared spectrum. NDIR can selectively detect gases such as CO, CO_2_, NOx, NH_3_, and HCl at room temperatures, but drawbacks encompass interference by water molecules and other gases [59]. In the electrochemical gas sensor, an electrode undergoes oxidation or reduction to detect the target gas, mostly via amperometry [60]. This type of gas sensor can be configured with two (working and counter electrodes) or three electrodes (with an additional reference electrode). Besides reacting with target analytes, the sensing material on the working electrode also serves as both an ion and electron conductor. For example, core‐shell CuO‐CeO_2_ nanoparticles loaded with carbon nanotubes (CNTs) and graphene oxide (GO) as the sensing material allow for the CO gas sensing by measuring the electric current, with CO gas reduced on the working electrode and the oxygen gas oxidized on the counter electrode [61]. This CNT‐loaded CuO‐CeO_2_ sensor exhibits a detection limit of 0.5 ppm with a response/recovery time of 3/6 s at 0.5 V for 100 ppm CO [62]. Although an electrochemical gas sensor exhibits a fast response time and high selectivity to specific gases, it relies on liquid/ionic electrolytes, which can evaporate or leak, resulting in baseline drift and increased calibration burden. Calorimetric gas sensors typically have either catalytic or thermometric configurations. Catalytic ones can measure heat changes caused by reactions of combustible gas on the sensor surface, but they need oxygen and can be contaminated. On the other hand, a thermometric gas sensor based on a heated sensing element to alter resistance or thermal conductivity upon interaction with gas molecules can detect gases such as hydrogen (187 mW·m^−^^1^·K^−^^1^) and methane (34 mW·m^−^^1^·K^−^^1^) with high thermal conductivity by comparing heat loss rates. However, ammonia (24.9 mW m^−^^1^ K^−^^1^) and carbon monoxide (26.6 mW m^−^^1^ K^−^^1^) are not detectable due to their similar thermal conductivities to air [60, 63]. Surface acoustic wave (SAW) sensors are based on the principle of measuring the changes in frequency and phase of the surface acoustic wave that propagates along a piezoelectric substrate and interacts with a gas‐sensitive layer. With SnO_2_ as the gas sensing layer, the SAW NO_2_ gas sensor exhibits a detection limit of 0.5 ppm NO_2_ and a response/recovery time of 12/15 s at 300°C to 10 ppm NO_2_, along with strong selectivity over CO, NH_3_, and H_2_S gases [64]. Chemiresistive gas sensors detect gases through resistance changes induced by adsorption‐driven modulation of charge carriers. In metal oxide semiconductors (MOS) sensors, the surface adsorbs oxygen molecules in the air, forming an electron depletion layer (EDL) for n‐type materials and a hole accumulation layer (HAL) for p‐type materials. Here, target gases react with adsorbed oxygen species and alter the Schottky barrier height, thus the conductivity [65]. For example, SnO_2_@ZnO chemiresistive sensors can detect as low as 5 ppm ammonia with a response/recovery of 4.05 /6.74 s in the range from 5 to 35 ppm [66]. But this type of sensor performance often relies on high operating temperature rather than room temperature operation. Compared with traditional gas‐sensing mechanisms, LIG‐based sensors uniquely overcome key limitations of conventional technologies by offering room‐temperature operation, mechanical flexibility, and tunable surface chemistry (Table 1).

**TABLE 1:** Comparison between LIG gas sensing and traditional gas sensing methods.

Method	Sensitivity	Operating temperature	Selectivity	Flexibility	Power consumption (mW)	Key limitations
Optical (NDIR)	ppb–ppt	RT	Good	Rigid optics	100–1000	Bulky optics, humidity interference
Electrochemical	ppm–ppb	RT	Good	Limited	<10	Electrolyte drift
Calorimetric	ppm	300°C–600°C	Poor	Rigid	>200	Non‐selective, heat loss errors
SAW	ppb	100°C–250°C	Poor	Rigid	10–100	Rigid substrates, alignment dependent
MOS Chemiresistive	ppm‐ppb	150°C–400°C	Moderate	Limited	50–300	Requires heaters, humidity sensitive
LIG‐based Sensor	ppb–sub‐ppb	RT	Tunable	Highly flexible/stretchable	<5–20	Requires surface engineering

*RT = Room Temperature, Ppb = Parts per Billion, Ppt = Parts per Trillion

### 2.1 Sensing Mechanism of LIG‐based Gas Sensors

**FIGURE 2:**
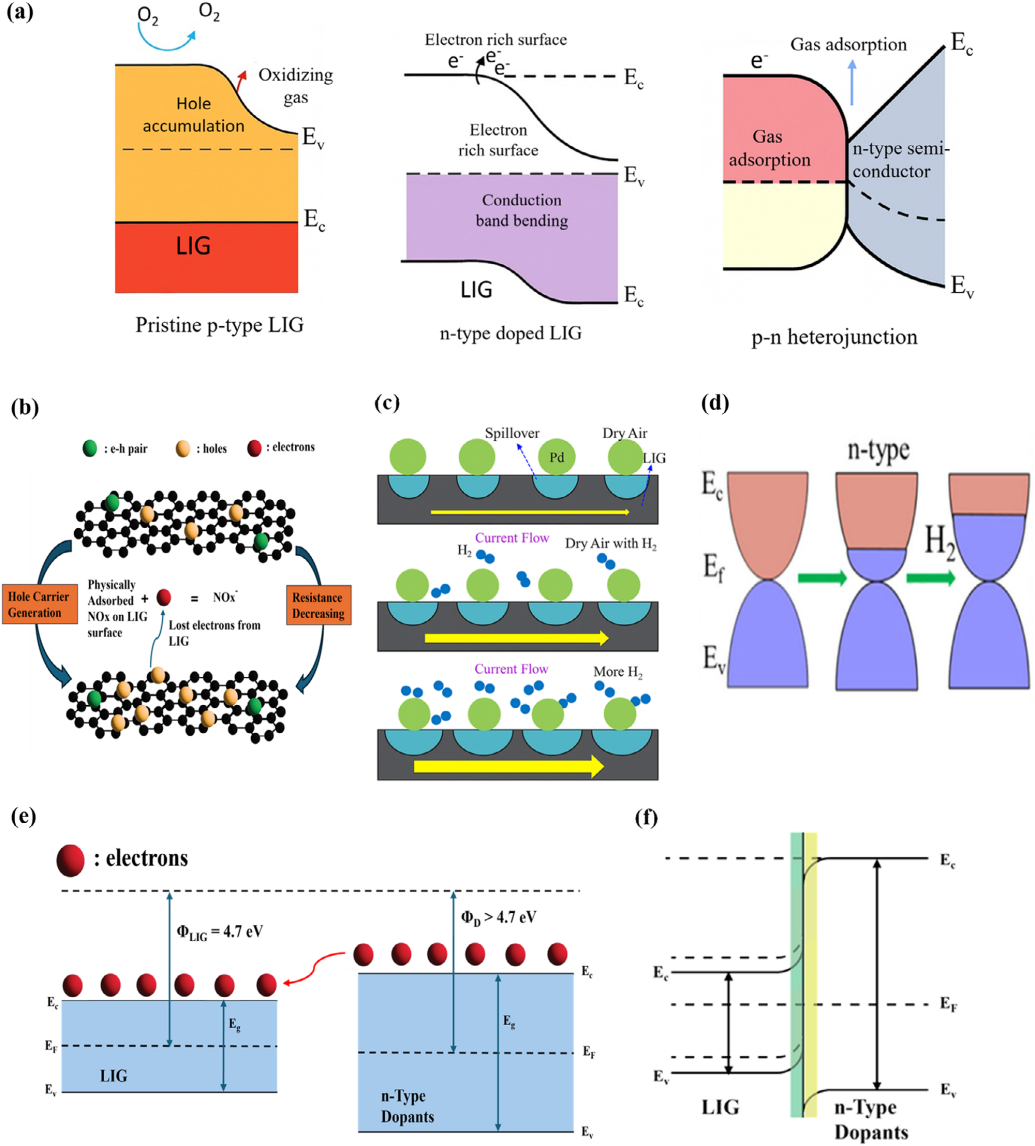
Gas sensing mechanism of LIG‐based gas sensors. (a)Schematic energy band diagrams showing charge‐transfer and band‐modulation mechanisms in pristine p‐type LIG, n‐type doped LIG, and LIG‐based p–n heterojunctions. (b) LIG donates electrons from its valence band to adsorbed NOx gas. Reproduced under the terms of the Creative Commons CC BY License 4.0 [67]. Copyright 2022, the Authors, published by Springer Nature. (c) LIG receives electrons in the presence of O_2_ and N_2_. Reproduced with permission [68] Copyright 2019, American Chemical Society. (d) Effect of H_2_ gas adsorption on LIG bands. Reproduced with permission [68]. Copyright 2019, American Chemical Society. Band diagrams of LIG and n‐type dopants in the LIG‐NO_x_ nanocomposite: (e) before and (f) after NO_x_ adsorption, showing the band bending phenomenon.

The gas sensing behavior of LIG can be rationalized through energy band modulation in pristine p‐type LIG, n‐type doped LIG, and LIG‐based p–n heterojunctions (Figure 2a). In p‐type LIG, adsorption of oxidizing gases induces electron withdrawal and surface hole accumulation, leading to pronounced band bending and resistance modulation [69]. In contrast, n‐type doping and p–n junction formation introduce electron‐rich surfaces and interfacial depletion regions, where gas adsorption perturbs the junction barrier and amplifies charge‐transfer‐induced resistance changes [70]. Chemiresistive sensing in pristine LIG relies on the charge transfer between p‐type LIG and gas molecules adsorbed on the active surface sites. This interaction results in changes in the resistivity of the sensing material when exposed to oxidizing or reducing gases [71]. For example, exposure to reducing NH_3_ molecules donates electrons to the hole‐rich LIG, partially compensating the majority hole carriers and thereby reducing the effective carrier density. This carrier compensation leads to an increase in resistance, with the relative resistance change rising from 5% to 30% as the NH_3_ concentration increases from 5 to 400 ppm [72]. On the other hand, exposure to oxidizing NO_x_ gas results in the extraction of electrons from the valence band of LIG (Figure 2b), leading to the accumulation of holes (main carriers) in the material. The extent of electron transfer is determined by the lowest unoccupied molecular orbital (LUMO) of the NO_x_ gas molecules, which is lower compared to other gases [67]. Consequently, a larger response is observed in LIG due to the greater number of transferred electrons. This unique behavior enables LIG to exhibit excellent selectivity specifically toward NO_x_ gases.

On the other hand, introducing O_2_ and N_2_ from the air during the fabrication of LIG can turn it into a p‐type material [68]. Further depositing palladium (Pd) nanoparticles as catalysts on the p‐type LIG by e‐beam evaporation can be exploited to sense H_2_ gas (Figure 2c). When H_2_ molecules are adsorbed on the Pd nanoparticles, the formation of the Pd hydride (PdH_x_) compound lowers the work function of Pd. As a result, the reduced Schottky barrier height between Pd and LIG increases the electron injection from Pd to the defect holes of LIG, increasing the conductivity of LIG for decreased resistance (Figure 2d).

Different kinds of gas‐sensitive nanomaterials introduced to the LIG‐based sensing layer enhance the interaction with the target gas molecules. For instance, drop‐casting n‐type material like MoS_2_ on LIG forms a p‐n heterojunction and a depletion region. The hydrophilic nature of LIG promotes the absorption of the MoS_2_ solution into the pores of LIG for enhanced contact and interaction. When gas molecules are adsorbed on the MoS_2_/LIG surface, they can cause charge transfer and influence the band bending between the n‐type MoS_2_ and p‐type LIG, resulting in a change in the electrical resistance of the junction by expanding the hole accumulation zone in the depletion region. N‐type MoS_2_ can also show p‐type behavior in the case of O_2_ gas adsorption. In synthetic air, O_2_ adsorption on MoS_2_ extracts electrons, inducing p‐type behavior to reduce resistivity. Adsorption of NO_2_ (with a higher binding energy of 220 kJ/mol over 42 kJ/mol for O_2_) [73]. further increases hole concentration, leading to a significant resistivity decrease. Upon returning to synthetic air from the NO_2_ environment, desorption of NO_2_ restores the resistivity to its initial state. In reducing the resistivity, compared to the MoS_2_ with 74% in the presence of 10 ppm NO_2_, the contribution of LIG of only 2%, which is negligible, so LIG mainly serves as an interdigitated electrode (IDE) here. This device can detect up to 1 ppm of NO_2_ gas at room temperature.

On the other hand, n‐type dopants like vanadium oxide (VO_x_) can be doped into LIG both with numerous oxygen vacancy defects and dangling bonds to facilitate the adsorption of oxygen molecules onto the composite at room temperature [54]. Doped VOx/LIG with defects enhances room temperature oxygen adsorption and NOx detection by increasing hole zones and decreasing resistance (Figure 2e,f).

Detection of low concentrations of gas molecules (∼1.2 ppb) at room temperature relies on a rational design of p‐n junctions (e.g., petal‐like rGO/MoS_2_ and ZnO/rGO core/shell structures) in the LIG composite [74]. Within rGO/MoS_2_ nanoflowers, the p‐type rGO sheets contribute to overall conductivity, whereas the n‐type MoS_2_ on the rGO sheets possesses multiple active sites with high selectivity to NO_2_.

Besides chemiresistive mechanism, intrinsic self‐heating and consequent thermoresistive behavior of LIG can be leveraged to sense gases with high thermal conductivity, such as N_2_ and CO_2_ from flue gas stream [75]. Under applied bias to LIG electrodes with an interelectrode gap of 20 µm, substantial joule heating occurs in LIG with a prominent effect at the microgaps where small LIG fragments bridge the electrodes. As a result, a localized hotspot is observed due to relatively high resistance. Upon exposing the electrodes, especially the microgap region, to target gases, a significant amount of generated heat is swept away by Newton's convection cooling mechanism to decrease the temperature and thus increase the resistivity of LIG upon gas adsorption. Conversely, removal or desorption of the gas reduces convective cooling, which allows the LIG to reheat and restore its baseline resistance. This thermoresistive mechanism gives an alternative way of gas detection, extending the utility of LIG beyond purely adsorption‐driven chemiresistive sensing.

### 2.2 Structural and Physicochemical Characterizations of LIG

**FIGURE 3:**
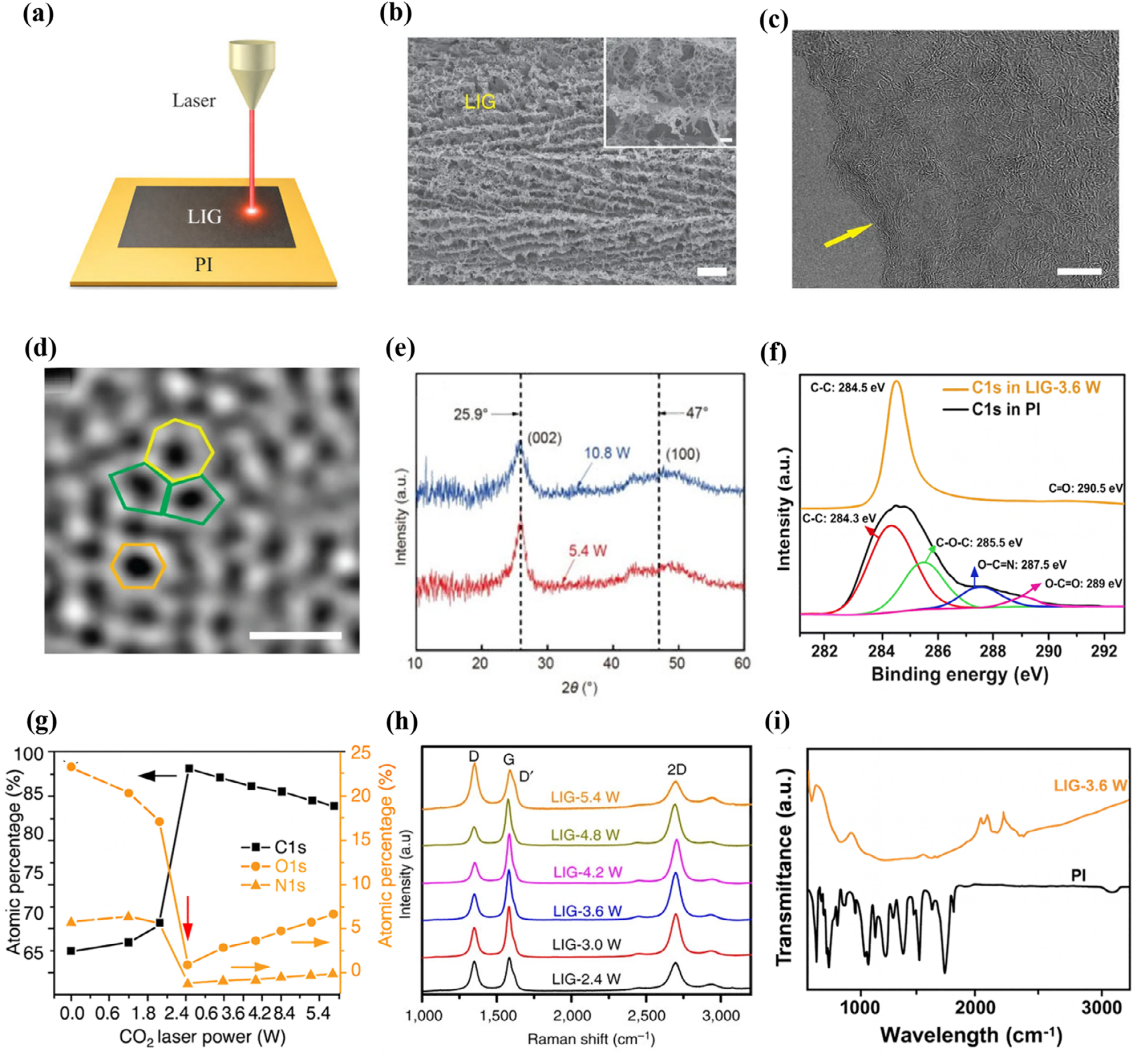
Characteristics of LIG. (a) Schematic of LIG formation on PI film. (b) SEM image of the LIG film fabricated at a laser power of 3.6 W. (c) High‐resolution transmission electron microscopy (HRTEM) image captured at the boundary of LIG flake (Scale 10 nm). (d) Cs‐STEM image taken at the boundary of a LIG flake (scale bar 2 nm). (b‐d) Reproduced with permission [76]. Copyright 2014, Springer Nature. (e) X‐ray diffraction (XRD) analysis of LIG fabricated at a laser power of 5.4 W and 10.8 W. Reproduced with permission [80]. Copyright 2021, Springer Nature. (f) XPS characterization of 3.6 W‐LIG films. (g) Atomic percentages of C, O, and N as a function of laser power obtained from XPS. (h) Raman spectra of LIG films obtained with different laser powers. (i) FTIR spectra of 3.6 W‐LIG and PI films. (f‐i) Reproduced with permission [76]. Copyright 2014, Springer Nature.

Characterization plays a crucial role in understanding and optimizing the properties of porous LIG for gas sensing applications. It also provides valuable insights into its structure, morphology, and performance. Laser processing parameters such as laser power, scanning speed, and image density significantly influence the film thickness, microstructure, morphology, and sheet resistance. SEM image of LIG films formed with laser power of 3.6 W from an infrared CO_2_ laser on commercial polyimide (PI) film (Figure 3a) shows a porous foamy visual aspect, which is a result of quick gas generation caused by the recombination of different types of atoms [76]^.^ (Figure 3b). These porous structures with increased surface area allow for improved accessibility and facilitate the infiltration of gas molecules into the active materials. The TEM image of LIG reveals few‐layered structures in thin flakes and mesoporous characteristics in thicker flakes. Moreover, the surface of the flakes displays ripple‐like wrinkled structures, enhancing the electrochemical performance of graphene‐based devices because of increased surface area and the creation of additional pathways for electron movements. Examination using high‐resolution TEM indicates that the nano‐sized ripples correspond to exposed edges of graphene layers (Figure 3c). These ripples are likely to form due to thermal expansion caused by laser irradiation. Moreover, the aberration‐corrected scanning TEM (Cs‐STEM) image illustrates the unique ultra‐polycrystalline nature of LIG flakes, with disordered grain boundaries arranged in a hexagonal lattice featuring a heptagon surrounded by two pentagons (Figure 3d). These pentagon‐heptagon pairs contribute to the curvature observed in the graphene layers, leading to the formation of the porous structure [77]. These inherent defects present in LIG offer promising possibilities for enhancing the electrochemical capacity of LIG and functionalizing the material in catalysis applications, as validated by theoretical calculations as well [78]. XRD peaks further confirm the presence of graphite order and the coexistence of layered graphene and amorphous carbon structure. The XRD patterns of LIG, even at different laser powers, display a prominent peak at 2*θ* = 25.90°, which corresponds to the (002) plane of LIG (Figure 3e) [79]. Calculated from the Bragg formula *n*·*λ* = 2*d*·sin*θ* (where *λ* is the wavelength, *d* is the interlayer spacing, *n* is an integer, and *θ* is the incident contact angle), the interlayer spacing of 3.42 Å indicates a high degree of graphitization in LIG [80]. Another peak observed at 2*θ* = 47° is indexed to (100) reflections, which are associated with the in‐plane structure [81]. However, this peak is not distinct and barely appears in the XRD patterns. XPS is used to analyze the presence and relative abundance of carbon and other elements in LIG samples. Besides the dominant C─C peak, the comparison in the high‐resolution C1s XPS spectrum of both the LIG film and the precursor material (PI) shows a significant reduction in the C─N, C─O, and C═O peaks from the PI to LIG (Figure 3f). This reduction indicates that LIG predominantly consists of sp^2^‐carbons, characterized by a high degree of graphitic carbon content. Laser power also plays a significant role in determining the atomic percentage of carbon, oxygen, and nitrogen in the resulting material. As the laser power increases from 2.4 to 5.4 W at a scan rate of 3.5 inches s^−1^, the atomic percentage of C experiences a sharp rise from the initial 71% to 97% in LIG, while a less than 3% decrease in N and O atoms (Figure 3g). In addition to laser power, lower scanning speeds and higher image resolution are critical for achieving the intended microstructure and reduced sheet resistance of the LIG. For example, a 30W CO_2_ laser operating at 10% of its maximal power, 11% of its scanning speed, and an image density of 6 can produce highly conductive LIG film from PI. Raman spectroscopy further provides valuable insights into the structural disorders with three distinctive peaks present in LIG: 1) the D peak at 1350 cm^−1^ corresponding to the disordered vibrations for assessing the defect characteristics, 2) the G peak near 1580 cm^−1^ from the E_2g_ mode of the doubly degenerate center to inform the symmetry and order of the structure, and 3) the 2D peak at 2695 cm^−1^ resembling the second‐order Raman peak of double phonon resonance in single‐layer graphene (Figure 3h). These 2D peaks reflect the stacking mode of carbon atom layers in graphene. Increasing laser power varies the intensity and half‐width of these characteristic peaks. FTIR spectra of the PI show characteristic peaks within the range of 1090–1776 cm^−1^ that correspond to the stretching and bending modes of the C─O, C─N, and C═C bonds in the PI structure (Figure 3i). In comparison, a broad absorption band spanning from 1000 to 1700 cm^−1^ is observed in LIG films formed at 3.6 W, indicating a significant alteration in the molecular structure and chemical composition caused by the laser scribing.

### 2.3 LIG‐Based NOx Gas Sensor Performance

Gas sensing plays a crucial role in monitoring and safeguarding both the environment and human health. By accurately detecting and quantifying various gases such as pollutants and hazardous substances in the air, gas sensors provide valuable data for assessing air quality, ensuring compliance with environmental regulations, and protecting individuals from harmful exposures. As nitrogen oxide (NOx) is detrimental to both people and the environment, reliable real‐time monitoring and early warning are of great significance. The exceptional physical and chemical characteristics of the 3D porous LIG render it an ideal contender for NOx gas sensing.

**FIGURE 4:**
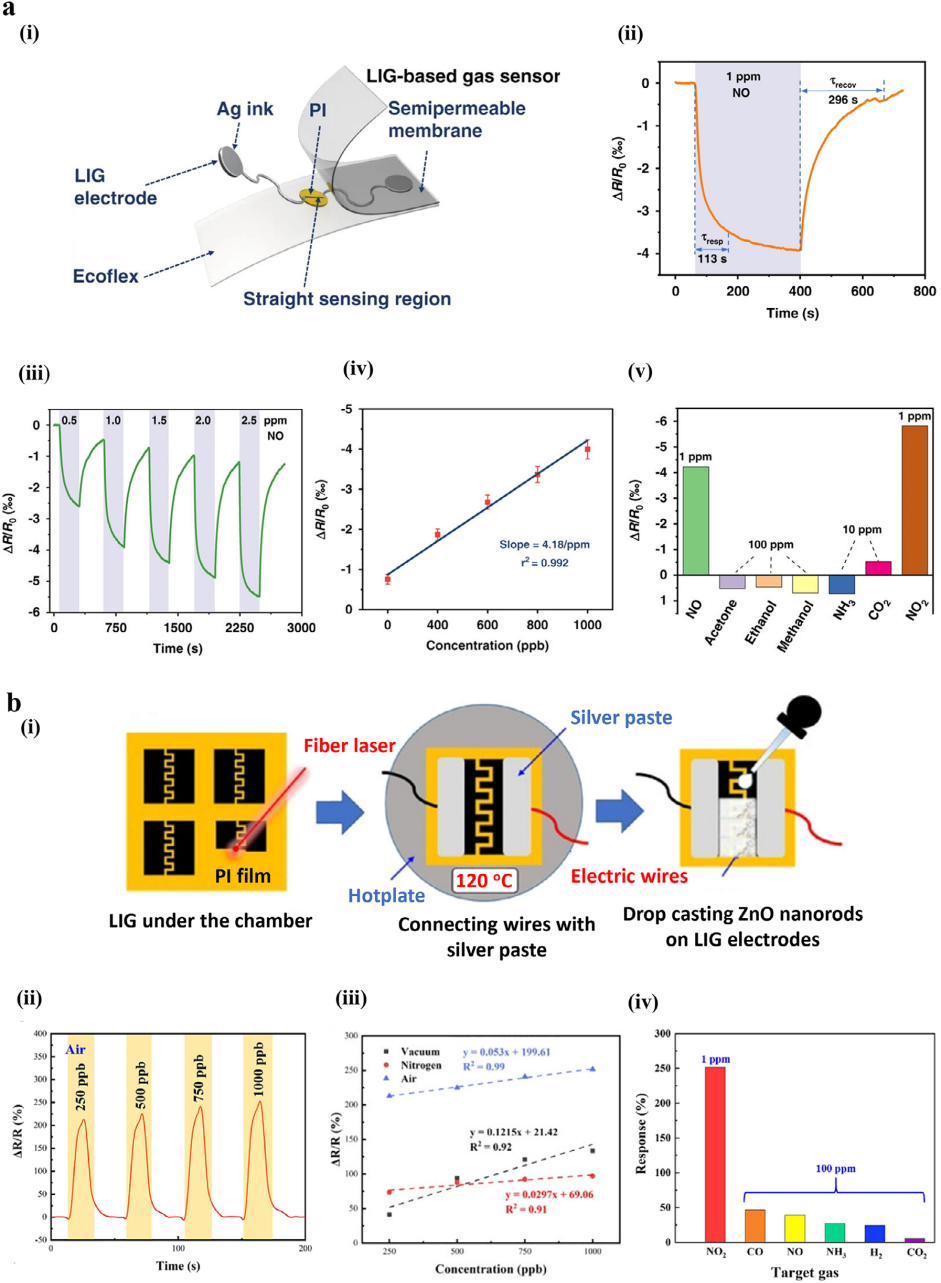
LIG‐based NOx gas sensor. (a) Moisture‐resistant, stretchable gas sensor: (i) Exploded view of the structural layout. (ii) Sensor response curve to 1 ppm NO. (iii) Dynamic response curves to NO concentration from 0.5 to 2.5 ppm. (iv) Calibration curve obtained from the sensor response to NO from 200 to 1000 ppb. (v) Selectivity test of the gas sensor to NOx over other interfering gases. Reproduced under the terms of the Creative Commons CC BY License 4.0 [67]. Copyright 2015, the Authors, published by Springer Nature. (b) ZnO nanorods/LIG‐based NO_2_ gas sensor (i) The fabrication steps for creating gas sensors based on ZnO nanorods and LIG. (ii) Response curves of LIG/ ZnO‐based gas sensors manufactured in air to NO_2_ in the range from 250 to 1000 ppb at room temperature. (iii) The correlation between the response of gas sensors on ZnO nanorods/ LIG and different concentrations of NO_2_. (iv) Selectivity of the ZnO nanorod/LIG‐based gas sensor to different target gases at room temperature. Reproduced with permission [83]. Copyright 2023, Elsevier.

Encapsulating the serpentine Ag/LIG sensing electrode on Ecoflex with semipermeable polydimethylsiloxane (PDMS) membrane results in a moisture‐resistant, stretchable NOx gas sensor (Figure 4a‐i) [67]. Increasing the laser power from 0.15 W to 0.6 W enhances the sensor response from 2.6% to 4.0% due to the higher specific surface area of the needlelike microstructure. However, a further increase in power to 1.2 and 1.8 W reduces the response to 3.10% and 2.20% (at 1 ppm NO), respectively, due to the collapsed hole‐like microstructure and reduced specific surface area at high power. At the optimized laser power of 0.6 W, the gas sensor also demonstrates a rapid response and recovery time of 113/296 s when exposed to 1 ppm NO at room temperature (Figure 4a‐ii). The sensor response increases from 2.6% to 5.5% as the concentration of NO is progressively increased from 0.5 to 2.5 ppm, indicating excellent dynamic response and recovery (Figure 4a‐iii). However, the incomplete recovery in the dynamic response can be attributed to the short recovery time used for rapid testing and the presence of residual charge carriers on the LIG repeatedly exposed to NO_X_. Strategies to address this include the use of elevated working temperature to enhance the desorption process of the absorbed gas molecules, which can be achieved through self‐heating (Joule heating) resulting from the significantly increased resistance of the sensing region compared to the electrode. The sensor response follows a linear relationship to NO from 200 to 1000 ppb, with a high correlation coefficient (R^2^) of 0.992 and a slope of 4.18 ppm^−1^ (Figure 4a‐vi). As the limit of detection (LOD) is defined as 3 times the root mean square (RMS) noise divided by the slope from the linear calibration curve, the LOD is calculated as 8.3 ppb. Compared to NO, more oxidizing NO_2_ causes a similar but more pronounced response in the sensor. Although the sensor response to NOx is relatively small, the SNR values for NO (463) and NO_2_ (679) are significantly higher than those of other gas sensors based on 2D nanomaterials. The highly porous LIG with a high specific area contributes to low contact resistance, which results in low noise and high SNR, further contributing to low LOD. The sensor also respond reliably to 20 ppb NO, showing a rapid 2.2% signal change with an SNR of 42.7, demonstrating its suitability for low‐concentration NO sensing. The sensor also shows a high selectivity to NO_X_ (1 ppm NO or NO_2_) compared to other interfering gases such as NH_3_, CO_2_, acetone, ethanol, and methanol (Figure 4a‐v).

Despite the use of porous LIG, long‐term gas sensing from the human skin with perspiration needs to explore breathable substrate materials. By facilitating the exchange of air and moisture, the breathable sensor enhances human comfort and avoids skin irritation. Spin coating block copolymer Pluronic F127/Phenol–formaldehyde resin(resol) self‐assembly on various knitted fabrics, followed by laser direct writing, prepares the intrinsically porous and breathable LIG. Further drop‐casting silver nanoparticles (AgNPs) on the LIG forms an Ag/LIG composite as breathable, flexible gas sensors [82]^.^ The Ag/LIG composite gas sensor exhibits quick response/recovery of 65/405 s and low detection limits of 6.5 ppb. Additionally, the Ag/LIG composite almost maintains the fabric breathability with a slight decrease in weight change measurements and vapor transmission rate compared to the pristine knitted fabric.

Because of the wide bandgap and high electron mobility [84, 85], zinc oxide (ZnO) nanostructures drop‐cast between the LIG interdigitated electrodes can be used for detecting NO_2_ gas (Figure 4b‐i) [83]^.^ Specifically, ZnO nanorods synthesized using a facile and low‐cost hydrothermal method at 80°C have a higher specific surface area (4.0768 m^2^/g) compared to ZnO synthesized at 120°C (2.4225 m^2^/g), 140°C (2.7144 m^2^/g), 160°C (1.6399 m^2^/g), and 180°C (2.6111 m^2^/g). Compared to a response of 41.25% to a 250 ppb NO_2_ for laser scribing in vacuum, the ZnO nanorod/LIG sensors fabricated in air and nitrogen exhibit responses of 212.92% and 251.71%, respectively (Figure 4b‐ii). The higher response of the sensor fabricated in air is attributed to the presence of larger and sharper pores in the LIG structure (thus larger surface area and more gas adsorption sites), in contrast to the sensor fabricated in vacuum and N_2_. Furthermore, the LIG electrodes created in an air medium exhibited a faster response/recovery time of 9.5/8.3 s and demonstrated higher repeatability compared to those fabricated in nitrogen or vacuum. The sensing layer formed in air medium also demonstrates high linearity (R^2^ = 0.99) compared to nitrogen/vacuum medium (0.91/0.92) (Figure 4b‐iii). The selectivity of the ZnO nanorod/LIG‐based gas sensor fabricated in air was impressive, with a response of 251.71% to 1 ppm NO_2_, significantly higher than responses to other gases at room temperature, indicating excellent selectivity for NO_2_ detection (Figure 4b‐iv). Long‐term stability testing over 30 days showed a total decrease in response of approximately 4.17% for the ZnO nanorod/LIG‐based gas sensor fabricated in air, confirming its excellent stability. This promising stability and selectivity make ZnO/LIG gas sensors suitable for integration with the Internet of Things for real‐time detection of harmful gases, showcasing their potential for practical applications.

**FIGURE 5:**
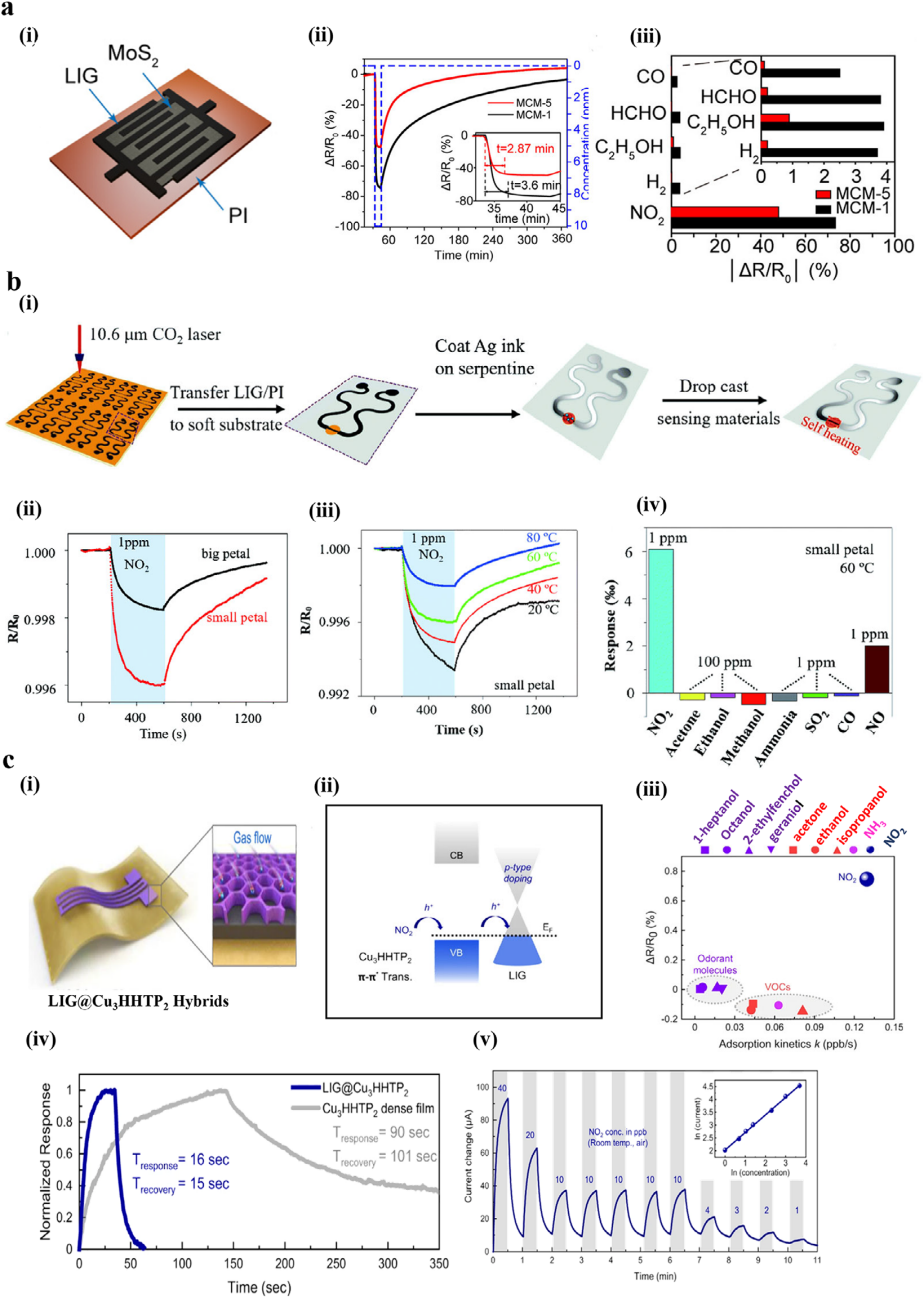
LIG‐based NOx gas sensor. (a) LIG gas sensor combined with flower‐like size tunable MoS_2_ nanopores. (i) Morphology‐controlled LIG‐based MoS_2 _(MCM) electrodes. (ii) MCM‐1 and MCM‐5 room temperature sensing characteristics, including dynamic response curves when exposed to 10 ppm of NO_2_ (inset). (iii) Selectivity of the sensors to different gases. Reproduced with permission [73]. Copyright 2020, American Chemical Society (b) LIG NOx sensor with self‐heating capabilities. (i) Steps for creating a flexible LIG gas detection platform. (ii) Response curves of LIG‐based rGO/MoS_2_ sensing platform with two different petal structures at 60°C to NO_2_ of 1 ppm. (iii) Response curve of rGO/MoS_2_ nanoflowers with tiny petals to 1 ppm NO_2_ at different temperatures resulting from self‐heating. (iv) The rGO/MoS_2_‐LIG gas sensor's selectivity for NO_2_ over a broad spectrum of other inferencing gaseous molecules at 60°C. Reproduced with permission [74]. Copyright 2020, Royal Society of Chemistry. (c) LIG‐based hybrid MOF gas sensor. (i) Schematic illustration of LIG@Cu_3_HHTP_2_ hybrid structure. (ii) Energy band diagram depicting the p‐type doping mechanisms in LIG@Cu_3_HHTP_2_ upon exposure to NO_2_. (iii) Evaluation of selectivity toward interfering gases, including NH_3_, various VOCs (ethanol, isopropanol, and acetone), and odorant molecules (1‐heptanol, 2‐ethylfenchol, octanal, and geraniol). (iv) Curve illustrating the response and recovery at room temperature for various ppb levels of NO_2_. (v) Contrast in response and recovery times between LIG@Cu_3_HHTP_2_ hybrids and dense Cu_3_HHTP_2_ films when exposed to 10 ppb NO_2_. Reproduced under the terms of the Creative Commons CC BY License 4.0 [86]. Copyright 2023, the Authors, published by Springer Nature.

Besides the nanomaterial itself, it is also vital to control its morphology to further optimize gas sensing properties at room temperature [87, 88]. The fabrication of the MoS_2_ nanospheres with varying morphologies relies on the use of ammonium molybdate tetrahydrate, thioacetamide, and different amounts (0, 40, 150, 500, and 800 mg) of sodium dodecylbenzenesulfonate (SDBS) surfactant, resulting in morphology‐controlled‐MoS_2_ (MCM) solutions named as MCM‐1, MCM‐2, MCM‐3, MCM‐4, and MCM‐5 [73]. After forming LIG on PI in ambient conditions, uniform coating of a 10 µL MoS_2_ solution within the interdigitated LIG electrode completes the fabrication (Figure 5a‐i). As the size of the MoS_2_ structure decreases (for samples from MCM‐1 to MCM‐5), the sensors' gas response decreases from ‐67.0% to ‐47.6%, while the response time reduces from 3.6 to 2.87 min (Figure 5a‐ii). The increase in surfactant amount changes MoS_2_ morphology from larger to smaller nanospheres (MCM‐1 to MCM‐4) and finally nanoplates (MCM‐5). MCM‐1 shows larger response times because of high‐energy binding sites (e.g., defects including vacancies and functional groups), whereas MCM‐5 shows a fast response time because of low‐energy binding sites (e.g., sp2‐bonded carbons), along with a recovery of over 95%. The gas response of the sensors to NO_2_ from 1 to 10 ppm follows a logarithmic relationship, with MCM‐1 and MCM‐5 exhibiting linear responses (R^2^ of 0.997 and 0.985, respectively). This difference in linearity is mainly because MCM‐1 has more defects and high‐energy adsorption sites, making its sensor response more consistent and linear. In contrast, MCM‐5 has fewer defects and higher crystallinity, leading to slightly more variable adsorption dynamics and response linearity. The sensors demonstrate low responses (<4%) to gases such as 1000 ppm of CO, C_2_H_5_OH, H_2_, and 10 ppm of HCHO, indicating their high selectivity toward NO_2_ (Figure 5a‐iii) [89]. MCM‐5 exhibits better selectivity for each interference gas compared to MCM‐1. Overall, the surfactant content effectively influences the morphological structures from nanospheres to nanosheets to intricately change the gas sensing capabilities by enhancing sensitivity through higher defect density and improving selectivity via enhanced crystallinity and thinner profile, highlighting how structural intricacies dictate gas sensor performance. In addition to surfactant‐tuned morphology, an *in‐situ* Au and MoS_2_ co‐decorated LIG (LIG/MoS_2_/Au) sensor can detect NO_2_ at an ultra‐low limit of detection of 3.6ppb at 90°C when compared with conventional metal oxides (operated at >200°C) [90]. The sensor shows a response of 11.4% compared to that of 3.4% from bare LIG to 1 ppm NO_2_ and a high SNR of 186.9. The selectivity to NO_2_ (1 ppm) is also confirmed from the dominant response compared to other interferents of ethanol, hexane, acetone, and NH_3_.

As these gas sensors often show slow response/recovery or even no recovery at room temperature, it is desirable to integrate heating elements to raise the temperature and enhance desorption. However, the performance of micromachined Si heating elements with a limited lifetime caused by chemical degradation deteriorates at high operating temperatures due to electromigration instability. By utilizing self‐heating capabilities from Joule heating, the LIG gas‐sensing platform removes the necessity for a separate heater [74]. The efficient Joule heating can be achieved by selectively coating the metal layer in the serpentine interconnect area to create a significantly smaller resistance in this region compared to the center sensing region (Figure 5b‐i). Coating the porous LIG with rGO/MoS_2_ or MoS_2_ nanomaterials substantially increases the response to 7% or 5%, respectively, marking a 20‐fold enhancement compared to the uncoated porous LIG sensing regions. Furthermore, after 6 min of exposure to NO_2_, the LIG sensing area coated with rGO/MoS_2_ or MoS_2_ exhibits a significant SNR of 482 or 285, respectively, indicating the effect of nanomaterials. To assess how width impacts gas sensor performance, rGO/MoS_2_ nanoflowers with varying petal sizes are selected. The smaller petal structure shows a higher response of 4% compared to the larger petal structure's response of 1.8% to 1 ppm NO_2_ at 60°C due to the even distribution of rGO/MoS_2_ and an increase in the surface area and formation of p‐n junctions on the interface (Figure 5b‐ii). This work introduces the angle of plateau (tangent of the response/recovery curve at the end of the adsorption and desorption) to measure the response and recovery of the sensor. The smaller the plateau angle, the faster the response and recovery. The plateau angle of 2° from the small‐petal rGO/MoS_2_ signifies a quicker response compared to the larger‐petal structures with a 3° plateau angle.

To investigate the self‐heating effect, the gas sensing behavior of the LIG‐based rGO/MoS_2_ sensor to 1 ppm NO_2_ is evaluated at different working temperatures ranging from 20°C to 80°C (Figure 5b‐iii). With an Ag/LIG serpentine interconnect, the temperature is modulated by applying voltage from 0 to 12V to induce joule heating. The temperature range under 100°C ensures stable ionosorption during charge transfer with MoS_2_ [91], as fewer MoS_2_ active sites become available for NO_2_ adsorption at temperatures higher than 100°C. In dynamic testing, the sensor demonstrates a rising response from 0.2 to 5.0 ppm NO_2_ with reversible behavior. The sensor also displays a steady response of 5% and quick response/recovery times of 360 s/720 s when subjected to 1 ppm NO_2_ for five successive cycles. The combination of highly porous LIG and rGO/MoS_2_ nanoflowers with a large specific area leads to minimal contact resistance, resulting in reduced noise with a high SNR. The small petal sensor showed a higher sensitivity with a slope of 7.49% ppm^−^^1^, compared with 5.42% ppm^−^^1^ for the larger‐petal and corresponding detection limits of 1.2 ppb and 2ppb, respectively. In comparison, ZnO nanorod/LIG and morphology‐controlled MoS_2_/LIG composite nanostructured gas sensors show detection limits ranging from only 1–10 ppm of NO_2_. Selectivity tests show a significant response of 5.1% to NO_2_ at 1 ppm concentration, with lower responses to low concentrations of NH_3_, SO_2_, and CO and higher concentrations of acetone, ethanol, and methanol due to the poor interaction with the sensing nanomaterials (Figure 5b‐iv). The responses to NH_3_, SO_2_, and CO of 1 ppm were small and showed an opposite direction due to their reducing characteristics [92]. Additionally, besides the common interfering gases, humidity is a significant concern for gas sensors operating at room or low temperatures in target application environments.

Doped LIG (DLIG) holds significant promise for NO_2_ detection, leveraging precise doping strategies to tailor its electronic properties for optimized response. By introducing dopants, surface chemistry can be fine‐tuned, enhancing its selectivity amidst complex gas mixtures. Notably, VO_X_‐doped LIG foam, comprising vanadium sulfide (V_5_S_8_)‐doped block copolymer and phenolic resin, has demonstrated remarkable performance, achieving an ultralow limit of detection (LOD) of 3 ppb at room temperature [54]. This exceptional sensitivity is attributed to the heterojunction formed at the interface between LIG and VO_X_, facilitating electron transfer processes upon exposure to oxidizing NO_X_ gases. The chemiresistive VO_X_/LIG gas sensor exhibits not only superior sensitivity but also higher selectivity and a reduced response recovery time. Specifically, the sensor showcases an impressive selectivity and response recovery time of 217/650s, underscoring its potential for real‐time NO_2_ monitoring applications. These findings highlight the efficacy of DLIG as a versatile platform for gas sensing, with implications spanning environmental monitoring, industrial safety, and public health initiatives.

In recent studies, hybrid semiconducting LIG has emerged as a promising platform for real‐time monitoring of trace levels of NO_2_ at room temperature. LIG integrated with Cu_3_HHTP_2_ metal‐organic frameworks (MOFs), termed as LIG@Cu_3_HHTP_2_, demonstrates exceptional performance in NO_2_ detection at ppb levels (Figure 5c‐i) [86]. This synergistic combination exhibited superior gas sensing capabilities, attributed to enhanced mass transport and adsorption capacity [93]. The fabrication process involved irradiating polyimide films with a pulsed 355 nm UV laser to generate UV‐induced graphene (UV‐LIG), allowing for programmable electrode patterning and miniaturization with a linewidth of 150 µm. The small thickness of 6 µm LIG without damaging PI and the low Young's modulus of 234 MPa contribute to the increased flexibility of the device. Directly grown Cu_3_HHTP_2_ MOFs on LIG sheets via a layer‐by‐layer (LbL) process can leverage LIG's chemical environment to facilitate MOF nucleation without additional functionalization steps, whereas traditional methods like drop‐casting of solvothermal solution or spin‐coating are mechanically unstable. LIG displays p‐type semiconducting behavior upon NO_2_ exposure, increasing hole carrier concentration in Cu_3_HHTP_2_ by withdrawing electrons from copper (Figure 5c‐ii). Moreover, the presence of functional groups and defects in LIG contributes to its gas‐sensing properties by inducing bandgap openings. Unlike the other examples discussed, here UV‐induced LIG introduces sp^3^ sites, vacancies, and edges that break sublattice symmetry and localize π‐electrons, effectively creating a finite transport gap and shifting the Fermi level. A small additional hole doped by NO_2_ generates a high chemiresistive gain and amplifies MOF selective uptake into a fast electrical signal.

The sensor exhibited high selectivity toward NO_2_ over interference gases such as volatile organic compounds (VOCs), odorant molecules, and ammonia, attributed to the uniform distribution of pore sizes in MOFs, enabling differentiation of guest molecule sizes and influencing adsorption rates (Figure 5c‐iii). LIG@Cu_3_HHTP_2_ sensor also shows a linear relationship from 40 to 1 ppb NO_2_ without requiring additional heat or light sources, demonstrating a sensitive theoretical LOD of 0.168 ppb (Figure 5c‐iv). Additionally, the sensor demonstrated rapid response and recovery times, with a remarkable response time of 16 s and recovery time of 15 s toward 10 ppb NO_2_, surpassing the performance of bare Cu_3_HHTP_2_ MOF sensors (Figure 5c‐v). The rapid response and recovery are due to the hierarchical porosity of LIG@Cu_3_HHTP_2_ to shorten the diffusion length, so time constants remain small (τ ≈ L^2^/D) even at the ppb level. In addition, open Cu sites and π‐stacked pores bind NO_2_ and p‐dope MOF/LIG immediately with moderate binding energy that allows rapid room‐temperature desorption. These findings underscore the potential of LIG‐based composite semiconducting materials as a versatile platform for sensitive and selective detection of NO_2_.

Tuning LIG enhances sensitivity to target gases due to the synergistic effects between LIG's high conductivity and the added materials' properties. Gas sensing properties (i.e., operating temperature, range of detection, LOD, response time, and recovery time) of the modified LIG gas sensor based on different materials and substrates for detecting NOx gas are summarized in Table 2. All the LIG‐based gas sensors except hybrid LIG@Cu_3_HHTP_2_ and ZnO nanorod/LIG composite sensors recover slowly than responding to the gas stimuli. Both of these sensors leverage a hierarchical porous structure that greatly enhances gas diffusion and interaction with the sensing material. This design mimics biological systems like the lungs, where a large surface area and efficient transport pathways allow for rapid gas exchange. In conventional sensors, the absence of such an optimized structure leads to slower gas diffusion and interaction, resulting in longer response and recovery times.

### 2.4 Other LIG‐based Gas Sensors

**FIGURE 6:**
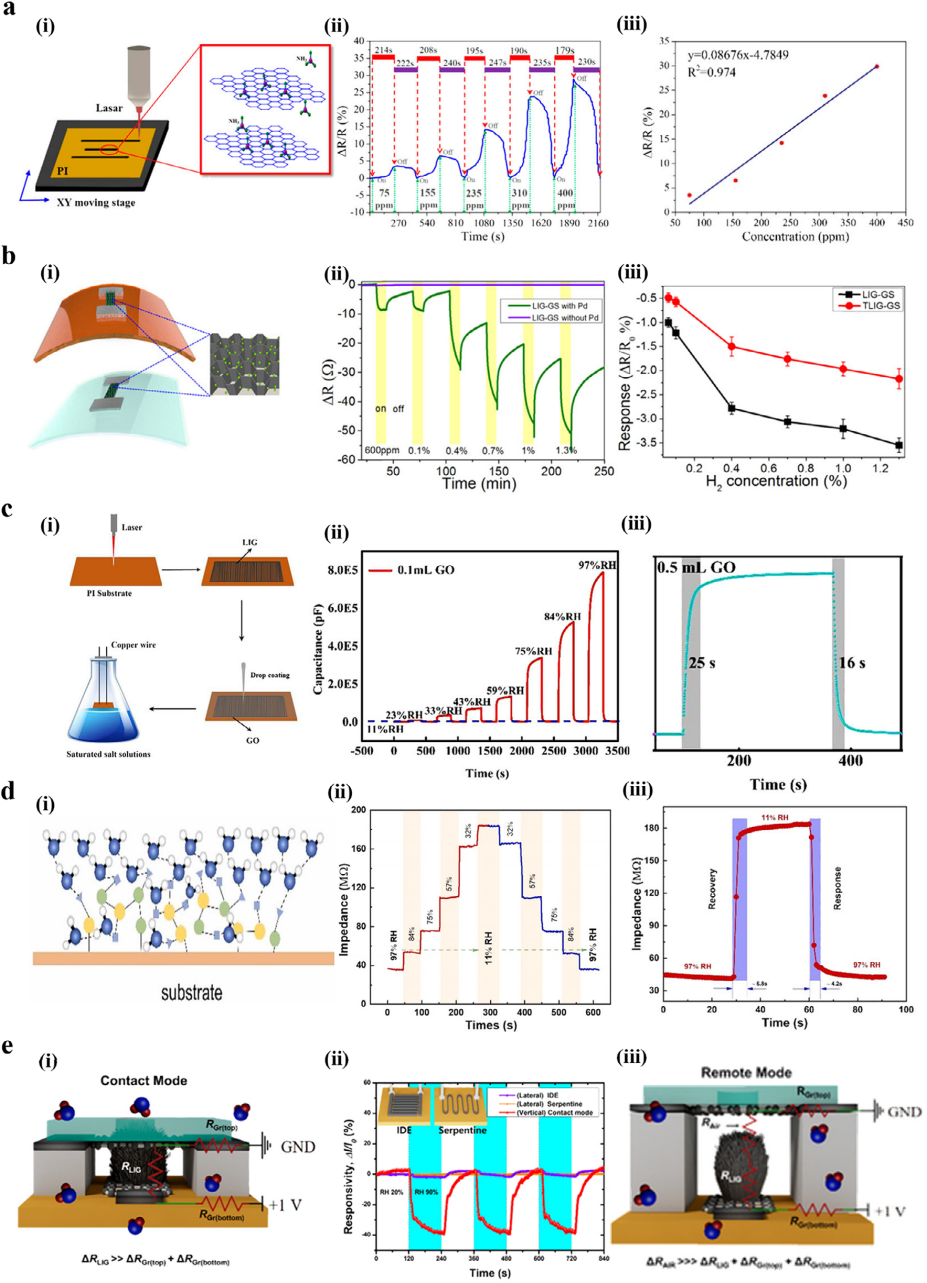
LIG‐based gas sensor for detecting NH_3_, H_2_, and humidity. (a) LIG‐based NH_3_ gas sensor. (i) Diagram illustrating the process of direct laser inscription. (ii) The sensor's response when exposed to different amounts of NH_3_ (75 to 400 ppm). (iii) The relative modifications in resistance experienced by the sensor when subjected to diverse concentrations of NH_3_ (75 to 400 ppm). Reproduced under the terms of the Creative Commons CC BY License 4.0 [72]. Copyright 2018, the Authors, published by MDPI. (b) LIG‐based H_2_ sensor. (i) Diagram illustrating the arrangement of palladium nanoparticles (Pd NPs) on graphene sheets modified with LIG (LIG‐GS) and transferred LIG (TLIG‐GS). (ii) Evaluation of the sensor's reaction to H_2_ gas at room temperature using LIG‐GS. (iii) Comparison of the sensor's response to various ppm of H_2_ gas. Reproduced with permission [68]. Copyright 2019, American Chemical Society. (c) GO/LIG humidity gas sensor. (i) Diagram depicting the process of preparing the humidity sensor. (ii) The time‐dependent behavior of the GO/LIG humidity sensor, at 500 mV and 500 Hz. (iii) Response/recovery time of sensitive layers using 1 mL of GO solutions. Reproduced with permission [95]^.^ Copyright 2020, Elsevier. (d) PEEK‐SP films humidity sensor. (i) Schematic of water molecules' adsorption and desorption by PEEK‐SP films. (ii) Response of PEEK‐SP sensors in the region of 11% to 97% relative humidity. (iii) Response and recovery time when switching between 11% and 97% RH. Reproduced with Permission [96]. Copyright 2023, Elsevier. (e) Vertical‐contact LIG nanotip humidity sensor. (i) Schematic of the humidity‐sensing mechanism of LIG nanotips operating in contact mode. (ii) Comparative response of vertical contact sensor with lateral‐contact interdigitated electrode (IDE) and serpentine patterns in the range of 20% to 90% RH variations. (iii) Schematic of the humidity‐sensing mechanism of LIG nanotips operating in non‐contact mode. Reproduced under the terms of the Creative Commons CC BY License 4.0 [97]. Copyright 2025, the Authors, published by Wiley‐VCH.

Tailoring the surface characteristics of LIG gas sensors shows considerable potential in detecting not only NOx but also a range of gases, including NH_3_, VOCs, humidity, H_2_, and several others. For example, developing three parallel porous 3D graphene lines on a PI tape can be used as an NH_3_ gas sensor [72]. One of the graphene lines serves as the NH_3_ sensing element, while the other two act as heating elements to enhance the desorption capabilities of the sensor (Figure 6a‐i). Graphene's large surface area makes it highly sensitive to NH_3_ molecules, allowing for their attachment and affecting electron transport. Even a single layer of graphene has the capability to detect molecular‐level gaseous species [98]. When graphene absorbs NH_3_ molecules, these molecules contribute electrons that combine with holes in the graphene conduction belt, resulting in a rise in electronic resistance in the sensing element. The 3D porous micro/nanostructures created by the laser direct‐written graphene lines facilitate the NH_3_ gas molecules' adsorption, thereby raising the sensor's sensitivity. The sensing performance of the ammonia gas sensor is evaluated under two conditions: with and without heating. The real‐time response/recovery behavior of the sensors to NH_3_ gas determined under concentrations ranging from 75 to 400 ppm shows that with increasing concentration, the normalized response of the sensor increases from 3.55% to 29.87%. The corresponding response/recovery times are 214/222 s to 75 ppm, 179/230 s to 400 ppm (Figure 6a‐ii). The resistance and NH_3_ concentration show a linear relationship with a sensitivity of about 0.087% ppm^−1^ (Figure 6a‐iii). Thus, the change in electrical resistance exhibits a positive linear correlation with ammonia gas concentration, while the relationship between response/recovery time and gas concentration is inverse. Thus, the variation in electrical resistance demonstrates a direct linear association with ammonia gas concentration, whereas the connection between response/recovery time and gas concentration is inverse. The cycling response test of the sensor in the presence of approximately 235 ppm NH_3_ gas indicates that the maximum normalized resistance change is around 15%. Nevertheless, the resistance does not go back to its initial value, and the sensor's recovery time increases with the increasing response cycles. Moreover, the resistance exhibits intensified drifting with the number of cycles, suggesting incomplete desorption of residual ammonia molecules from the sensing element. To address this issue, applying voltage to the heaters to elevate the temperature and promote desorption can enhance the cycling stability. To establish the optimal heating temperature in proximity to the sensing element and enhance heating effectiveness, the heating temperatures are adjusted within the range from 50°C to 90°C (to 235 ppm NH_3_). At 50°C, the graphene gas sensor shows incomplete desorption with a final resistance change rate of about 0.40%, while at 70°C and 90°C, the resistance change rates approach zero, indicating complete ammonia gas desorption and a return to the initial resistance value. However, the time required to raise the temperature to 90°C and cool down is lengthy, affecting the overall detection time. Therefore, a heating temperature of 70°C provides a shorter detection time.

Electrochemically deposited polypyrrole (PPy) on LIG (PPy@LIG) chemiresistive sensing layer further demonstrates room temperature detection of NH_3_ at a lower concentration of 1 ppm [99]. Uniformly coated PPy on LIG modulates the entire percolation network and undergoes reversible de‐doping upon exposure to NH_3_ via acid‐base/charge transfer by reducing hole density and increasing resistance. PPy@LIG shows a sensitivity of 2.13% per 100 ppm, 14 times higher than bare LIG. Though the sensor exhibits a moderate response time of 318 s, the recovery time of 2775 s is significantly long, due to slow desorption and re‐protonation kinetics, plus diffusion through a swollen polymer matrix. The sensor also exhibits high selectivity to NH_3_ of 0.75% at 5 ppm compared to a negligible response of 0.12% to 100 ppm CO, 0.05% to 1000 ppm H_2_, 0.10% to 20 ppm ethanol, 0.08% to 10 ppm benzene, and ‐0.19% to 100 ppm NO_2_. A recent work on polyaniline/LIG (PANI/LIG) outperforms the previous LIG‐based NH_3_ sensor with faster kinetics and high signal amplitude even at room temperature [100]. The PANI/LIG chemiresistor shows a response of 68% to 60 ppm with response/recovery time of ∼53/167 s and a limit of detection of 14.6 ppb. Here, the short recovery time is due to the conformal PANI on porous LIG, which minimizes the diffusion length and allows fast desorption and re‐protonation at room temperature. The sensor also shows a discernible response on selectivity of 0.25% to 5 ppm NH_3_ compared to 0.05% to 100 ppm CO, 0.03% to 1000 ppm H_2_, 0.05% to 20 ppm ethanol, 0.03% to 10 ppm benzene, and 0.06% to 100 ppm NO_2_.

3D LIG sensing platform decorated with PdNPs inspired by the turbinate structure found in dogs' olfactory systems has been demonstrated for H_2_ detection at room temperature [68]. LIG‐gas sensor (LIG‐GS) on a PI substrate, as well as the transferred LIG‐gas sensor (TLIG‐GS) on a polyethylene terephthalate (PET) substrate, are developed (Figure 6b‐i). To impart sensitivity and selectivity to H_2_ gas, Pd nanoparticles were uniformly coated on the full device through an e‐beam evaporation process, resulting in a Pd film thickness of 2 nm. The sensing mechanism operates through the catalytic impact of Pd nanoparticles on crystal defects within the biomimetic LIG turbinate‐like microstructure. This turbinate‐like microstructure includes a curved, layered, and folded structure, which maximizes the effective surface area and facilitates the straightforward adsorption and release of nonpolar H_2_ molecules compared to flat or less structured LIG. The sensor displayed a nearly linear sensing response in relation to the concentration of hydrogen gas. The gas reactions observed in both the LIG‐GS and TLIG‐GS (Figure 6b‐ii, iii) reveal that the original LIG without Pd did not show any response to H_2_ gas. Interestingly, the sensing response curves for LIG‐GS and TLIG‐GS devices differed, with TLIG‐GS showing an average sensitivity approximately 20% lower than that of LIG‐GS. This decrease in sensitivity can be attributed to the graphene microstructure crack occurred during the mechanical transfer process. Notably, the turbinate microstructure of LIG demonstrates 2–3 times higher sensing performance compared to the flat LIG. Both LIG‐GS and TLIG‐GS sensors exhibit reliable sensing performance in repeated H_2_ gas experiments. The LIG‐GS and TLIG‐GS both demonstrate high selectivity toward H_2_ compared to no response to 5 ppm NO_2_ and 25 ppm NH_3_ gases at room temperature. This selectivity is attributed to the specific reaction between hydrogen gas and Pd, forming PdHx, thereby conferring high selectivity of the fabricated Pd sensors toward H_2_ gas.

Complementing the PdNPs turbinate structure, deposition of pre‐synthesized platinum nanoparticles on LIG (Pt/LIG) leverages the same H_2_ disassociation and spill over but highlights high nanoparticle dispersion on porous LIG, resulting in the detection limit at the ppb level at room temperature due to LIG's high surface area and Pt catalytic activity [101]. The Pt/LIG sensor shows a response of 15.4% to 10,000 ppm, response/recovery time of 295/495 s to 1,000 ppm H_2_, limit of detection of 200 ppb, and cyclic stability over 50 cycles. The sensor also shows high selectivity with 2.57% to 400 ppm H_2_, exceeding other interferents such as acetone, benzene, ethanol, methanol, SO_2_, and toluene. Both PdNPs/LIG and Pt/LIG sensor responses are greatly influenced by the humidity content of the environment. At low to moderate humidity levels, H_2_ gas response can increase due to H_2_ dissociation/spillover via added surface O/OH and proton shuttling, but at higher humidity levels (>70%), water film forms to block Pd or Pt active sites and slow H_2_ dissociation. Strategically applying hydrophobic/H_2_‐permeable barriers (ZIF‐8 or fluoropolymer/parylene coatings) to block water films and adding brief self‐heating/duty‐cycled purge to desorb H_2_O can stabilize sensor response [102].

Humidity can be sensed by pairing LIG interdigitated electrodes with a hydrophilic graphene oxide (GO) layer (2mg/ml dispersion) (Figure 6c‐i) [95, 103]. GO films with different thicknesses and varying the size of the interdigital electrode can tune the sensor's performance. Different relative humidity (RH) levels from 11% to 97% can be generated using different saturated salt solutions, including LiCl, NaCl, KCl, MgCl_2_, Ch_3_COOK, K_2_CO_3_, NaBr, KNO_3_, and K_2_SO_4_. The study evaluates the effectiveness of humidity sensors with and without a graphene oxide film by monitoring ambient humidity levels. The sensors are operated based on capacitive sensing principles, measuring changes in capacitance of the sensing layers across a humidity range of 11% to 97% RH and a frequency range from 20 Hz to 10 kHz. The GO/LIG humidity sensor's ability to attract water is primarily due to the presence of the GO film. The capacitance of the GO sensor at different humidity levels (Figure 6c‐ii) demonstrates that higher humidity levels lead to increased capacitance, indicating the absorption of water molecules, whereas a decrease in capacitance signifies desorption. The process of water molecule adsorption can be simplified into several stages at higher humidity levels (43%‐97%). Meanwhile, the absorption of water molecules is confined to a single stage at low humidity levels (11%‐43%). Initially, at low humidity levels, water molecules attach to active sites on the GO surface using double hydrogen bonds; with increasing adsorption, they switch to single hydrogen bonds; at this point, the molecules become ionized due to the electrostatic field. At this stage, the sensitivity *S* follows a linear relationship with the relative humidity RH (*S* = AB·RH, with A and B as the experimental parameters) [104]. At high RH levels, water molecules enter the GO film layers, and ionic conduction becomes predominant. Consequently, changes in capacitance accurately reflect the quantity of water molecules adsorbed, representing the humidity level. Therefore, the sensitivity maintains an exponential relationship at high humidity (*S* = A (1‐e^−B·RH^)) [104]. To evaluate the reversibility of humidity sensors to reflect the absorption and desorption processes of water molecules, the sensor is exposed to air with RH levels of 33%, 59%, 75%, and 97%, and then switched back to an RH of 11% several times. In the limited range of humidity changes, the absorption and release of water molecules happen quickly, with a small hysteresis. The fast response and recovery of the sensor contribute to the low hysteresis of the sensor. Overall, the findings showed that the sensor displayed consistent cyclic performance, a broad range of responsiveness, and heightened sensitivity across all relative humidity (RH) levels tested.

The influence of sensing film thickness is further explored by varying the volume of GO solution used for drip‐coating. When 0.5 mL of GO solution is used, the porous structure of the sensitive film is completely covered, while at 1 mL, the film appears flatter with fewer wrinkles. Measurements using 0.2 mm electrodes show differences in response and recovery times. The best response is obtained with a GO solution of 0.5 mL, while the shortest recovery time (2 s) is observed with a 0.1 mL volume (Figure 6c‐iii). This could be attributed to water molecules immersing less into deeper layers of the GO film and a decrease in proton jump. Therefore, better sensing performance could be achieved with a 0.2 mm electrode size and a GO solution volume of 0.1 mL. In another study on LIG/GO‐based humidity sensor, the response and recovery times increase with increased thickness of GO due to the increased concentration of GO from 0.07 mg/mL to 2 mg/mL [105].

To overcome diffusion‐limited kinetics in thicker GO/LIG films, an alternative LIG‐IDE platform using a polymerized PEEK hygroscopic film (PEEK‐SP) improves humidity sensing with low hysteresis and long‐term stability. The LIG interdigital electrodes (LIG‐IDE) on flexible poly(ether‐ether‐ketone) (PEEK) substrates, along with impedance‐type hygroscopic film achieved through PEEK sputtering and polymerization methods [96]. The PEEK‐SP film sensing mechanism is based on hydrophilic groups like carboxyl (‐COOH) and aldehyde (‐CHO), which adsorb water molecules (Figure 6d‐i). The hysteresis behavior of the humidity sensor was investigated at a frequency of 200 Hz, observing impedance changes during both RH increase (from 11% to 97%) and decrease (from 97% to 11%) (Figure 6d‐ii). Notably, the impedance values from the RH rise and drop tests are closely aligned, indicating a hysteresis of approximately 6.5% suggesting the sensor built on PEEK‐SP films exhibited effective recovery and minimal hysteresis. The time required for response and recuperation was determined by alternating humidity settings between 11% RH (LiCl) and 97% RH (K_2_SO_4_), resulting in a response time of approximately 4.2 s and a recovery time of around 6.8s (Figure 6d‐iii). The device shows higher sensitivity and reduced response and recovery times compared to the recent flexible humidity sensors (Table 3). Additionally, the study examined the durability of the PEEK‐SP humidity sensor by exposing it to three sealed glass bottles with diverse saturated solutions (11% RH, 57% RH, and 97% RH) for a month. Impedance stability was tested every 3 days under varying RH conditions, yielding consistently stable impedance responses.

**TABLE 2:** Gas sensing properties of LIG‐based gas sensors for detecting NO_2_ gas.

Material	Substrate	Operating temperature	Range of detection(ppm)	Limit of detection (ppb)	Response time	Recovery time	Reference
LIG‐MoS_2_	PI	150°C	2‐5	0.317	NR	NR	[94]
LIG‐CuO	PI	140°C	NR	9.8	400s	800s	[85]
LIG‐Ag/ZnO	PI	100°C	NR	5.6	400s	1100s	
Ag/LIG	Textile	RT	0.5‐2.5	6.5	40s	291s	[82]
ZnO nanorod/ LIG	PI	RT	0.25‐1	NR	9.5 s	8.3 s	[83]
LIG	Ecoflex	RT	NR	4	134 s	388 s	[67]
rGO/MoS_2_‐LIG	PI	60°C	1	1.2	360s	720s	[74]
LIG‐MoS_2_	PI	RT	10	NR	172s	NR	[73]
LIG@Cu_3_HHTP_2_	PI	RT	1‐40	0.168	16s	15s	[86]

^*^RT = Room Temperature, ^*^PI = Polyamide Films, ^*^NR = Not Reported

Although the PEEK‐SP film‐based LIG humidity sensor shows low hysteresis and low response/recovery time of less than 10s, lateral‐contact IDE geometries still have limitations in response due to partially exposed films. A vertical‐contact LIG nanotip geometry can overcome this challenge by fully exposing the sensing interface [97]. This vertical contact LIG nanotip can operate in two modes: a contact mode and a non‐contact mode (remote). In vertical contact mode, vertically aligned LIG nanotips form completely exposed ultrathin current pathways between top and bottom graphene electrodes (Figure 6e‐i). The humidity modulates the nanotip resistance R_LIG_ and dominates the total sensor response ΔR_LIG_ ≫ ΔR_Gr(top) +_ ΔR_Gr(bottom)_. The vertical contact mode shows 40.8% responsivity, temperature robustness in the range from 24°C to 42°C, and stability of 90% over 7 days. Moreover, the vertical nanotip contact design improves the responsivity by 266 and 114‐fold compared to the conventional LIG‐based IDE and serpentine patterned sensor due to surface‐dominated electronic transport in the nanotip region, where adsorption‐induced charge modulation is maximized by the fully exposed, tapered, porous morphology (Figure 6e‐ii). In remote contact mode, the top and bottom electrodes are separated by air. In the presence of humidity, field ionization occurs due to intense electric fields, and H_2_O molecules are ionized to H_2_O^+^ ions and free electrons via quantum tunneling without direct contact of the electrode to the molecule (Figure 6e‐iii). This method substantially increases responsivity to 14 000% and response/recovery time <1 s across an RH range from 20% to 90%.

**TABLE 3:** Humidity sensing properties of different flexible humidity sensors.

Materials	Test‐range	Sensitivity	Response/Recovery	Reference
Paper/LIG	23‐85%RH	0.5 Ω/ %RH	95s/637s	[106]
AuNP @NC	6‐97%RH	4.9 pF/ %RH	11/43s	[107]
GO/LIG	11‐97% RH	9150 pF/ %RH	25/16s	[95]
SPEEK Nanofiber	30‐80%RH	0.07078 pF/ %RH	75s/75s	[108]
NaCl Modified Paper	5.6‐90%RH	45.72 A/A %RH	1208s / 537s	[21]
GO/LIG	0‐97% RH	1800 pF/ %RH	16s / 9s	[105]
GO	11‐97% RH	1.113 Ω/Ω %RH	2s / 35s	[109]
PEEK/SP	11‐97%RH	1700 kΩ/ %RH	4.2s/6.8s	[96]

Beyond sensitivity, detection limit, and response dynamics, the long‐term operational stability of LIG‐based gas sensors remains a critical consideration for practical deployment. A number of studies have reported baseline drift, humidity‐induced signal fluctuations, and partial recovery loss during prolonged cycling, particularly under room‐temperature operation and humid conditions. These issues primarily originate from strong adsorption of water molecules on oxygen‐rich LIG surfaces, slow desorption kinetics of target gases, and irreversible trapping of charge carriers at defect sites. To mitigate these challenges, surface engineering, physical isolation (introducing membranes), modulation of working parameters, and humidity compensation have been introduced [110, 111, 112]. By compensating humidity using the deep back propagation (BP) neural network, the NO_2_ sensor maintains quantitative accuracy (<1.37% error, mean square error = 0.0087) across wide humidity variations, overcoming the typical deviations of >20% in the uncompensated operation. The addition of polydimethylsiloxane (PDMS) membrane to the NO_x_ sensor blocks water adsorption while allowing NO_x_ molecules to diffuse to the sensing surface, thereby maintaining the NO_x_ response under varying humidity conditions from 0 to 80%RH. In contrast, the sensor without a PDMS membrane becomes humidity‐dominated and forms a surface water layer that suppresses NO_x_ adsorption and destabilizes the electrical baseline, leading to a near‐undetectable NO_x_ signal under humid conditions [113]^.^ Self‐heating via Joule heating in LIG enables accelerated desorption and baseline recovery without external heaters, thereby improving cycling stability. In addition, heterostructure engineering and controlled doping introduce moderate adsorption energies that balance sensitivity and reversibility. Emerging approaches further incorporate signal decoupling and data‐driven correction techniques to compensate for drift under varying environmental conditions. Collectively, these strategies can help enhance stability along with sensitivity optimization.

### 2.5 Strategies with Membranes in Gas Sensors

**FIGURE 7:**
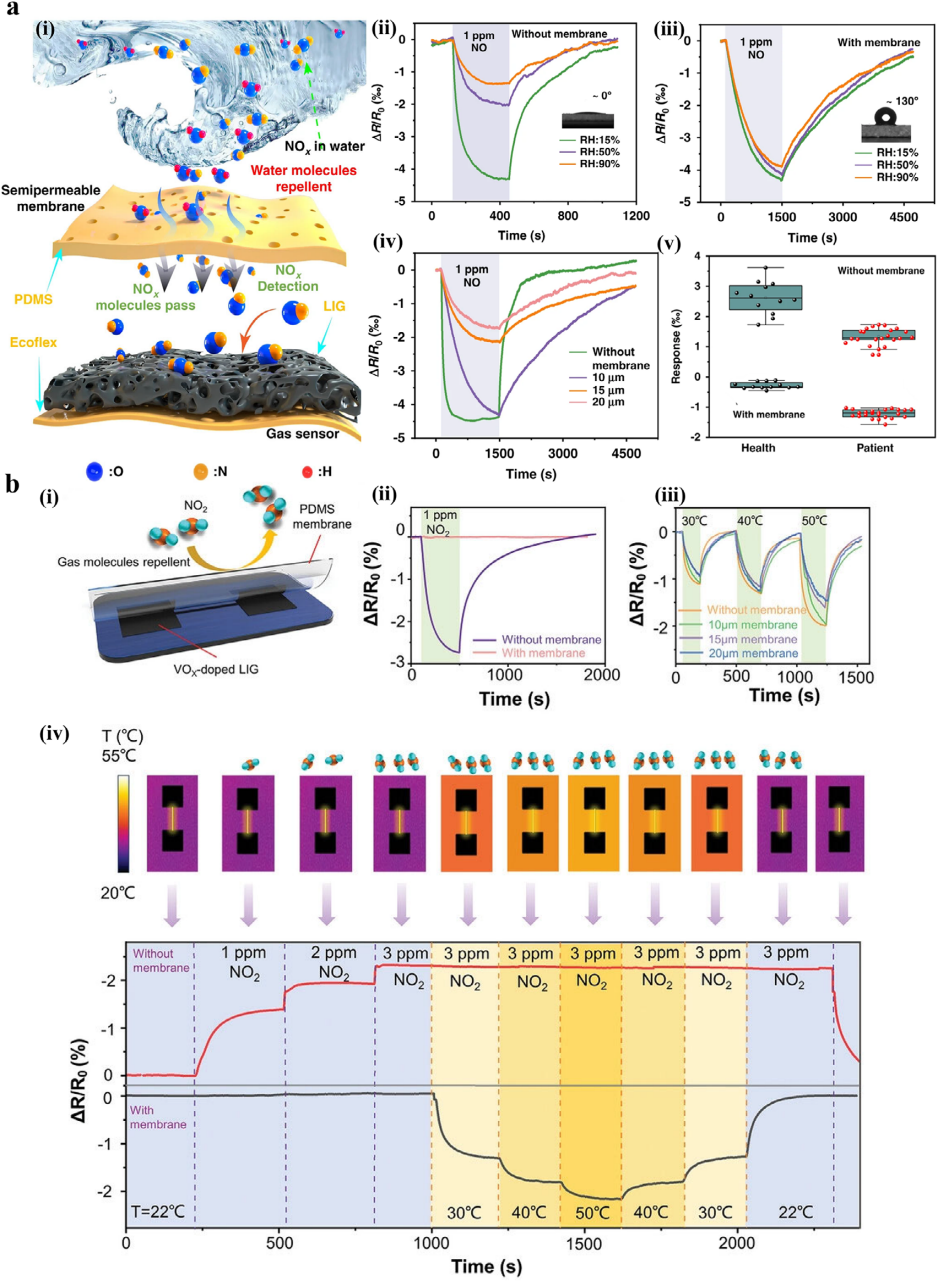
Membrane deployment strategies for LIG‐based gas sensors. (a) Moisture‐resistant LIG‐based gas sensor. (i) Depiction demonstrating humidity‐free gas sensing under wet conditions. Response of gas sensors in different relative humidity environments (ii) without and (iii) with a semipermeable PDMS membrane, with the water contact angle shown in the inset. (iv) Influence of semipermeable membrane thickness (ranging from 10 to 20 µm) on the response of gas sensing. (v) Gas sensor's response comparison when exposed to exhaled breath samples from patients with respiratory illnesses and healthy volunteers, both with and without the semipermeable membrane. Reproduced under the terms of the Creative Commons CC BY License 4.0 [67]. Copyright 2022, the Authors, published by Springer Nature. (b) Demonstration of the VOX‐doped LIG decoupled gas sensor. (i) Schematic of the enclosed sensor designed to prevent the passage of gas molecules. (ii) Response of the sensor in the presence and absence of the enclosed membrane to 1 ppm NO_2_. (iii) Response with different thicknesses of encapsulation to temperatures of 30°C, 40°C, and 50°C. (iv) Application of the enclosed VO_X_‐doped LIG sensor alongside the unenclosed version, both operated at 50°C, to fully isolate the influences of NO_2_ gas and temperature. The top visuals display the alterations in gas concentration and temperature. Reproduced under the terms of the Creative Commons CC BY License 4.0 [54]. Copyright 2023, the Authors, published by Wiley‐VCH.

The high relative humidity within exhaled breath creates a notable obstacle for gas sensing [114]. This challenge primarily stems from the significant influence of water molecule adsorption on the sensitive layer's reactivity. This impact is particularly pronounced for hydrophilic LIG. Though the superhydrophobic coating offers excellent water resistance, the sensor suffers from reduced gas permeability and durability issues [115]. However, introducing a semipermeable PDMS membrane onto the LIG sensing region can create an effective route for NOx diffusion via the siloxane backbone (Si‐O) [116]. Simultaneously, water and aqueous components are repelled, attributed to the reduced surface energy stemming from the methyl group (Si‐CH_3_) (Figure 7a‐i). When comparing the responses of the LIG‐based gas sensor both without (Figure 7a‐ii) and with (Figure 7a‐iii) the semipermeable PDMS membrane at 1 ppm NO under RH levels spanning from 15% to 90%, the role and impact of water resistance become evident. In contrast to the sensor lacking a semipermeable PDMS membrane, which experiences a substantial decline in response from 4% to 1.3% as RH escalates from 15% to 90% (Figure 7a‐ii), the sensor incorporating hydrophobic PDMS (with a water contact angle of around 130°) displays minimal fluctuations (Figure 7a‐iii). However, the utilization of the semipermeable PDMS membrane increases response and recovery times from 130/350 s to 890/2810 s due to the reduced diffusion rate of gas molecules through PDMS [117, 118]. This concern can be alleviated by operating at higher temperatures, utilizing self‐heating. As the operating temperature increases from 25°C to 60°C, response/recovery times decrease from 890/2810 s to 403/591 s. Furthermore, the gas sensor with a semipermeable membrane also presents satisfactory dynamic response/recovery across various operating temperatures. To expedite diffusion, a thinner PDMS layer is advantageous. In the same vein, the gas sensor with the thinnest 10 µm PDMS membrane sustains a substantial response of approximately 4%, akin to the one lacking PDMS (Figure 7a‐iv). The gas sensor with and without the permeable membrane was used to analyze human breath samples to potentially diagnose respiratory conditions like asthma or Chronic obstructive pulmonary disease (COPD). In the analysis, breath samples from 23 asthma or COPD patients and 12 healthy volunteers were evaluated. Notably, the response values from the gas sensor void of the semipermeable PDMS membrane exhibit notable dispersion, ranging from 1.73% to 3.61%, even within the group of healthy volunteers. This variability is likely attributed to the influence of relative humidity (RH), where the positive response of the sensor is prompted by water molecule adsorption on the sensitive layer. In contrast, the sensor integrated with the semipermeable membrane effectively mitigates the humidity's impact, leading to a significantly narrower response range of ‐0.12% to ‐0.46% provid (Figure 7a‐v). This augmentation in classification accuracy is evident as the sensor's response values for patients with respiratory diseases are approximately 4.8 times greater than those of their healthy counterparts (Figure 7a‐v).

Unlike the aforementioned semipermeable PDMS membrane, another study indicates that a thick, impermeable PDMS membrane can serve as an enclosing layer for the VO_X_‐doped LIG sensor [54]. This membrane creates a mass transfer barrier for NO_2_ while allowing heat transfer, resulting in a gas‐insensitive temperature sensor (Figure 7b‐i). Inclusion of a 10 µm‐thick PDMS membrane around the sensor results in a notably reduced reaction to NO_2_ at room temperature and 80% RH (Figure 7b‐ii). Remarkably, the swift transmission of heat within this thick PDMS membrane has minimal impact on temperature sensing, leading to an insignificant variance when compared to the unenclosed sensor's response (Figure 7b‐iii). Moreover, the encapsulated sensor can be paired with the unencapsulated sensor, operated at an elevated temperature through self‐heating, in order to entirely disengage the effects of temperature and NO_X_ gas (Figure 7b‐iv).

### 2.6 Next‐generation Standalone LIG Gas Sensor for Remote Applications

**FIGURE 8:**
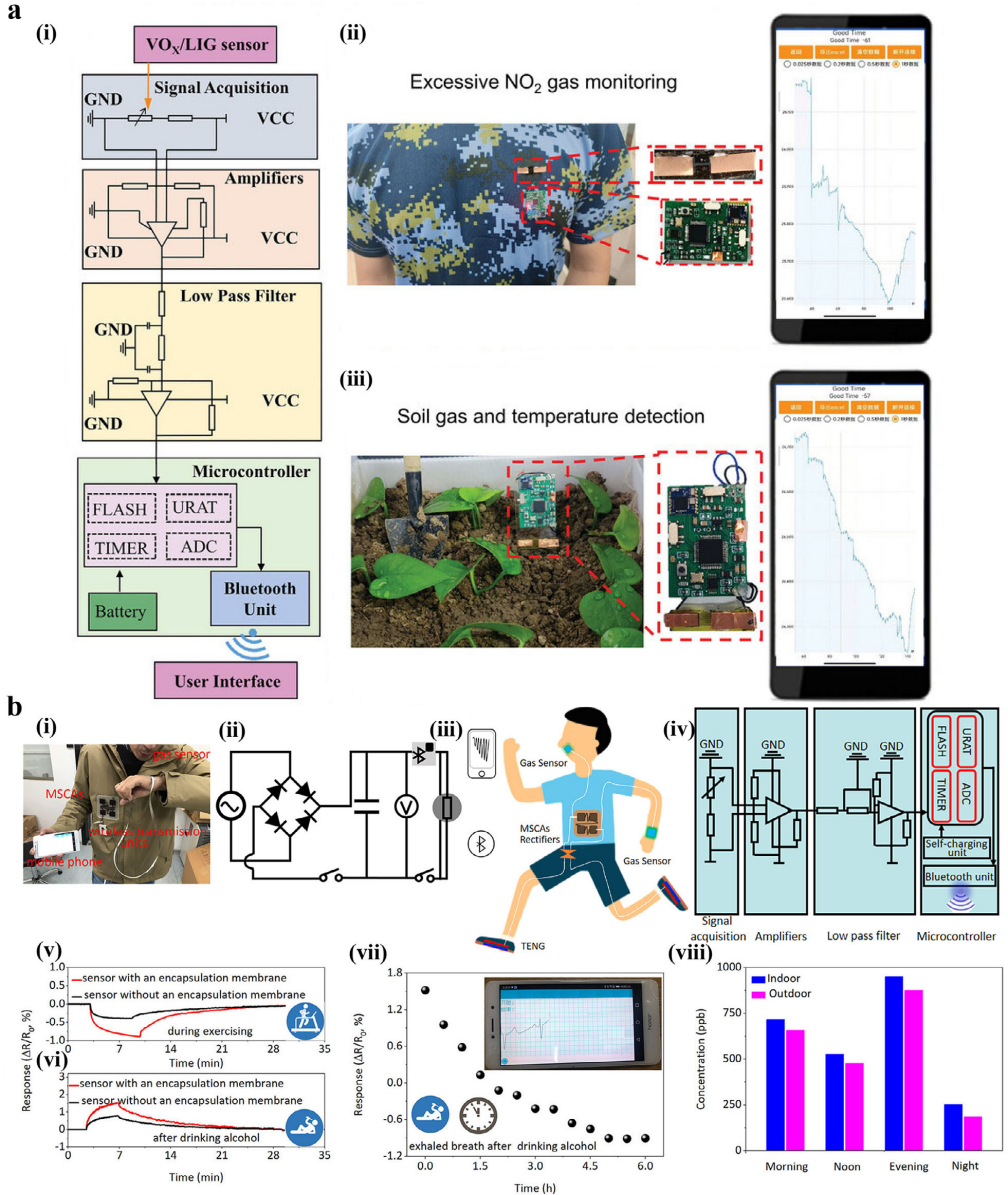
Illustration of next‐generation decoupling gas sensor and stand‐alone gas sensor (a) The development of a comprehensive remote environmental monitoring system utilizing the VO_X_/LIG sensor technology. This system comprises: (i) designing the circuit for remotely monitoring real‐time NO_2_ levels within the immediate vicinity of a person, and (ii) detecting soil gas and temperature for use in smart farming practices. Reproduced under the terms of the Creative Commons CC BY License 4.0 [54]. Copyright 2023, the Authors, published by Wiley‐VCH. (b) Illustration of an independent gas detection system utilizing a stretchable nanocomposite for environmental surveillance. (i) Image, (ii) electrical circuit schematic, and **(iii)** diagram illustrating the self‐powered integrated platform affixed to the clothing worn by the person. (iv) The design of the circuitry for the wireless transmission module. The response of the standalone sensing system, both with and without encapsulation, to the exhaled breath of the individual, (v) both during physical activity and (vi) after alcohol consumption. (vii) The real‐time monitoring of the individual's exhaled breath after alcohol consumption. (viii) Utilizations of the gas detection platform for monitoring indoor and outdoor nitrogen dioxide (NO_2_) levels emitted from vehicle exhausts during various times of the day, all powered by self‐recharging units. Reproduced with permission [58]. Copyright 2023, American Chemical Society.

The LIG sensor offers the potential for creating a comprehensive remote monitoring system for both the environment and human health. Wireless technologies provide a remote power supply, and data communication will play a crucial role in this remote monitoring system [119, 120, 121]. Different types of wireless communication technologies enable these devices to exchange data without cables. Such technologies include Bluetooth using electromagnetic waves [122], ZigBee using a mesh network method [123], Radio frequency identification (RFID) based on radio waves to identify short‐range and contactless information [124], and near‐field communication (NFC) based on the principle of electromagnetic induction [125, 126], for data transmission. Among them, Bluetooth offers the benefit of a broad transmission range and a fast speed, with a maximum transmission distance of about 100 m (compared to RFID of 15m, NFC of 20cm) and a top transfer rate of 24 Mb/s [121]. Bluetooth communication can be integrated into microcontroller modules, which are commercially available, thus making them a significant player in the wireless sensor market. In general, the sensor signal collected via a signal acquisition unit is amplified via an amplifier and filtered to restrict the high‐frequency noise via a low‐pass filter. This signal is then sampled in a digital format by the Analog to Digital Converter (ADC). A microcontroller sends the processed data to the Bluetooth module via Universal Asynchronous Receiver‐Transmitter (UART) protocol. In the end, the Bluetooth module sends the data wirelessly to a mobile application or website. The integrated sensor system enables real‐time data analysis, interpretation, and transmission to a centralized controller or monitoring station. This capability allows for continuous monitoring of environmental conditions without any human intervention, which is particularly useful for remote or inaccessible areas. For example, the wireless VOx/LIG sensor can be applied to precision agriculture applications [54]. It aids in continuous data to the Artificial Intelligence‐based models to monitor essential parameters such as the presence of harmful gases and soil temperature. This data‐driven prediction can inform farmers about the optimal conditions for crop growth, help prevent disease outbreaks, and enhance overall agricultural productivity. The VOx/LIG sensor connected to a Bluetooth‐enabled microcontroller powered by a battery (Figure 8a‐i) changes its resistance when exposed to gas and temperature stimuli and thus changes the voltage across the signal acquisition unit. After being amplified and filtered, the signal was then wirelessly transmitted to a mobile application for NO_2_ monitoring (Figure 8a‐ii) and decoupled soil gas and temperature detection (Figure 8a‐iii).

To ensure the sustainable operation of wireless LIG sensors, a strategy that provides continuous power is essential. The primary method of powering wearable sensors is using batteries such as lithium‐ion batteries due to their high energy efficiency [127, 128]. However, this type of battery always has a big size and heavy weight, which hinders its further development in wireless wearable devices. Besides, its rigid property limits the comfortable attachment to the skin. To address these issues, more efforts are needed to develop flexible and stretchable batteries based on wireless power transfer or even energy harvesting technologies. A recent development of a standalone self‐powered gas sensing system eliminates the usage of a battery, thereby decreasing the weight and price drastically [58]. The wireless system was mechanically powered by a Triboelectric Nanogenerator (TENG). Triboelectricity has recently gained a lot of attention due to its high electric output capacity with a minimal mechanical force applied by leveraging the triboelectric nature of two contacting materials. Here, the TENG‐powered charging device can supply continuous power for gas sensors and a Bluetooth module, which can wirelessly transmit the processed data to the user interface (Figure 8b‐i‐iv). When operated by a volunteer running at frequencies of 2.0 Hz, the triboelectric nanogenerator (TENG) produces a peak output voltage of 51.8 V, which increases with the increase in frequency. The study presents a proof‐of‐concept for a stretchable gas sensing platform placed on the skin, powered by human motion during intense exercises. This platform can effectively monitor both exhaled breath from individuals and NO_2_ levels from car exhaust. To maintain sensor stability and accuracy, exhaled breath is collected through a PET face mask before being introduced to the sensor inside a gas bottle using a PET pipe. The platform, with or without a gas‐permeable PDMS membrane, enables continuous analysis of breath constituents during various activities like exercise (Figure 8b‐v) and drinking alcohol (Figure 8b‐vi). Continuous monitoring of breath after consuming alcohol offers a straightforward yet effective method for assessing the impact of alcohol (Figure 8b‐vii). The stretchable gas sensor is designed to track NO_2_ gas emissions wirelessly and consistently from car exhaust throughout the day and night. It focuses on NO_2_ as the primary gas of interest, with measured responses of ‐1.064% in the morning, ‐0.841% at noon, ‐1.340% in the evening, and ‐0.513% at night, correspond to NO_2_ concentrations of 715 ppb in the morning, 527 ppb at noon, 950 ppb in the evening, and 252 ppb at night (Figure 8b‐viii).

Despite recent advances in wireless and self‐powered sensing platforms, several industrial barriers still limit the widespread deployment of gas sensors in real‐world applications. Conventional metal‐oxide, polymer, and electrochemical gas sensors often require high operating temperatures, involve complex microfabrication, and rely on rigid and brittle substrates, all of which increase manufacturing cost and reduce long‐term reliability. Their performance typically suffers from baseline drift, humidity interference, slow response/recovery dynamics, and difficulty in maintaining calibration over extended outdoor operation. Furthermore, integrating these sensors into distributed IoT networks demands low‐power electronics, high mechanical robustness, and scalability to large‐area or unconventional surfaces, which current platforms struggle to provide. From a commercial perspective, the dominant barriers include: (i) high per‐unit cost, making large sensor networks economically impractical; (ii) limited flexibility for wearable or conformal deployments; (iii) short operational lifetime under fluctuating environmental conditions; and (iv) incompatibility with automated roll‐to‐roll manufacturing, which is essential for mass production in environmental, agricultural, and industrial monitoring.

Laser‐induced graphene (LIG), however, offers a compelling pathway to overcome these constraints and enable true commercial‐grade, distributed gas‐sensing systems. First, LIG can be patterned directly on polymer substrates in a single‐step, mask‐free, room‐temperature process, allowing ultra‐low‐cost, high‐throughput fabrication compatible with roll‐to‐roll printing. Its intrinsically porous 3D graphene architecture provides high surface area, rapid gas diffusion, and tunable surface chemistry, enabling fast response and high sensitivity without external heating. The excellent mechanical compliance of LIG, arising from its hierarchical microcracks and interconnected graphene networks, supports bendable, stretchable, and wearable form factors that maintain stable electrical properties under deformation, addressing a major unmet need for industrial IoT and human‐centric monitoring. Additionally, LIG can seamlessly integrate with wireless modules, flexible energy harvesters (TENGs), and low‐power microcontrollers, enabling battery‐free or battery‐assisted operation with minimal system overhead. Collectively, these features position LIG as a strong candidate for scalable commercialization: it reduces manufacturing cost by orders of magnitude, enhances environmental stability, supports large‐area deployment, and accelerates digital integration—ultimately bridging the gap between laboratory demonstrations and industrial‐scale sensing infrastructure.

## 3 Conclusion and Future Perspectives

**FIGURE 9:**
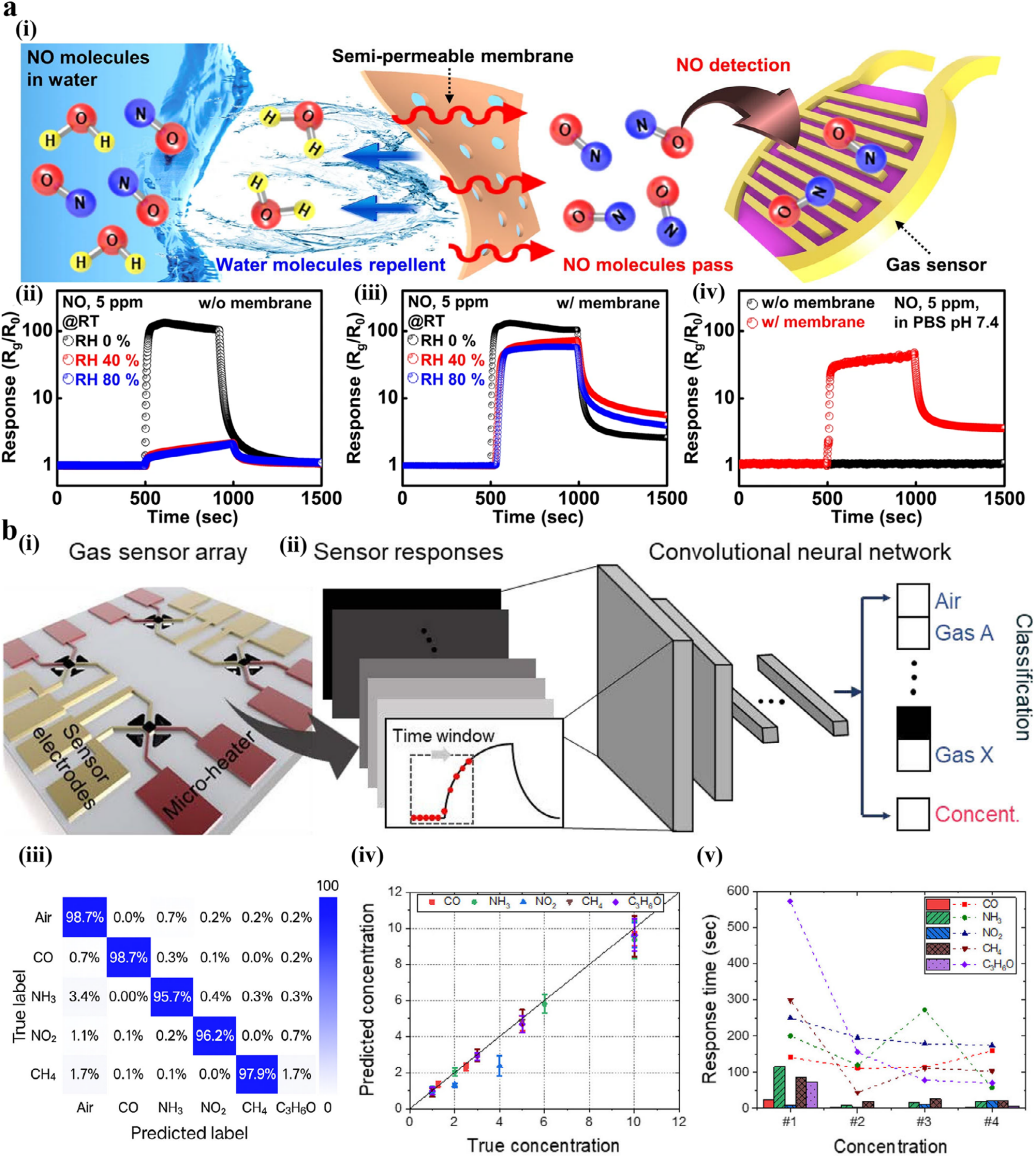
Strategies for robust and intelligent gas sensing. (a) Macrophage‐derived NO detection. (i) Semi‐permeable membranes made of polydimethylsiloxane (PDMS), approximately 50 µm thick, permit the selective passage of gas molecules while serving as a hindrance to water molecules. Assessments of the electrochemical reactions of integrated gas sensors both (ii) without and (iii) with a semipermeable membrane (approximately 50 µm thick) when exposed to 5 ppm of NO gas at different relative humidity levels (represented by colors: black for 0%, red for 40%, and blue for 80%). (iv) The electrical characteristics of integrated gas sensors in the absence (depicted in black) and presence (indicated in red) of semipermeable membranes while submerged in a phosphate‐buffered saline (PBS, pH 7.4) solution. Reproduced under the terms of the Creative Commons CC BY License 4.0 [116]. Copyright 2020, the Authors, published by Springer Nature. (b) Machine‐learning‐enabled gas sensing. (i) Scalable semiconductor metal oxide (SMO) gas sensors using glancing angle deposition (GLAD) with micro‐heaters. (ii) A Convolutional Neural Network (CNN) is employed for real‐time gas detection and measurement. It uses sensor response features that capture data from a defined time window. Reproduced with permission [129]. Copyright 2022, American Chemical Society.

LIG serves as a highly sensitive and selective active material for continuous gas monitoring applications. The LIG gas sensors are cost‐effective and scalable, and provide real‐time monitoring, thus making them suitable for public health monitoring devices. However, these sensors have not yet been explored in the realm of implantable devices, which could open up another set of diverse opportunities to monitor vasodilation, neurotransmitters, respiratory system. A major issue of implantable devices is the requirement of multiple surgeries to implant or explant devices into the body. In the last decade, transient electronics have been proposed, which mitigate the secondary operation by dissolving themselves with time passively or actively. In the recently reported transient, flexible gas sensor based on a single‐crystal silicon nanomembrane (SiNMs) (Figure 9a‐i) [116]. bioresorbable magnesium electrodes are deposited on a poly(lactic‐co‐glycolic) acid (PLGA) substrate, whereas the biocompatible elastomer PDMS is utilized as a semi‐permeable membrane for providing stable gas sensing performance in humid or wet conditions. The PDMS membrane offers hydrophobicity and gas permeability due to methyl groups on its surface, reducing surface energy and siloxane backbones and forming a pathway for NO diffusion. Comparative experiments without (Figure 9a‐ii) and with (Figure 9a‐iii) the membrane show a slight reduction in responsiveness, but electrical sensitivity remains high. Without the membrane, there is almost no response, even with minimal moisture. Similar results are observed during immersion in aqueous media, where the membrane enables stable detection in a PBS solution, while no signals are detected without it (Figure 9a‐iv). Most of the gas sensors focus on detecting individual gases (NOx, NH_3_, or H_2_) rather than detecting multiple gas systems, similar to the human nose. To overcome this, an electronic‐nose (E‐nose) system collecting multiplex responses of various gases at different temperatures can decipher the sensor data to deconvolute multiple gases using machine learning techniques (Figure 9b‐i) [129]. The semiconductor metal oxide‐based gas sensor array is employed to categorize types of gases and predict their concentrations by deploying a convolutional neural network (CNN) to decipher the gas response patterns. With the training and testing data of the gas sensor array for six types of gases (including the air condition), an accurate gas type classification consistently achieves a high classification accuracy (98.06% across all conditions). Furthermore, simultaneous gas concentration predictions are made with an average error of 10.15% (Figure 9b‐ii, iii). Additionally, the minimum response time required to identify gas types for CO, NH_3_, NO_2_, CH_4_, and C_3_H_6_O is notably shorter, clocking in at 1, 8, 5, 19, and 2 s, respectively. This highlights the ability to classify gas types much faster compared to the typical response time of 112, 57, 174, 44, and 70 s for CO, NH_3_, NO_2_, CH_4_, and C_3_H_6_O, respectively (Figure 9b‐iv).

To achieve a truly deployable wireless sensing system, two primary development strategies have emerged: (1) incorporating active wireless communication modules into conventional circuits or (2) developing passive or self‐powered sensing platforms. Each strategy presents distinct advantages and engineering constraints. Integrating active wireless modules (e.g., Bluetooth, ZigBee) enables real‐time, high‐bandwidth data transmission, typically with communication ranges spanning 10–100 m for Bluetooth, up to 100 m – 1 km for ZigBee mesh networks, and tens to hundreds of meters for sub‐GHz protocols. This feature makes them suitable for distributed sensor networks in agriculture, industrial safety, and environmental monitoring. However, these modules impose significant power demands due to continuous radio‐frequency (RF) transmission, requiring either larger batteries or efficient power‐management circuits, which complicates system miniaturization. Additionally, active transmitters necessitate careful electromagnetic shielding and impedance matching to prevent signal distortion, especially when integrated with soft or stretchable substrates such as LIG‐based devices.

In contrast, passive wireless approaches, such as NFC, RFID, or solar‐energy‐assisted platforms, offer ultra‐low power operation and can even eliminate batteries entirely. NFC communication is typically limited to a few centimeters, while passive RFID tags operate over ∼0.5–10 m, depending on antenna geometry and reader power. These constraints can restrict use in large‐area monitoring but significantly simplify the electronics and reduce long‐term maintenance requirements. However, passive systems are associated with intrinsic challenges, including short read ranges, susceptibility to environmental RF interference, limited energy availability under low‐light or intermittent illumination (in the case of solar harvesting), and the need for optimized antenna–sensor co‐design to maintain signal fidelity under mechanical deformation. Solar‐powered systems depend strongly on illumination intensity, spectral conditions, and surface orientation, which may vary across use environments.

In conclusion, this review explores recent advancements in LIG‐based gas sensor technology, covering its operational principles, manufacturing techniques, and performance in various gas detection applications. It discusses the gas‐sensing mechanisms of LIG and its nanocomposites, including fabrication methods and key characteristics such as sensitivity, selectivity, response time, and detection limits. The review also underscores the potential of wireless and standalone gas sensing platforms for wearable sensors. It concludes by outlining future prospects for LIG gas sensors, highlighting their potential to revolutionize gas sensing technology and address environmental and human health challenges.

## Conflicts of Interest

The authors declare no conflicts of interest.

## Data Availability

The authors have nothing to report.
